# Synthesis and Thermal, Photophysical, Electrochemical Properties of 3,3-di[3-Arylcarbazol-9-ylmethyl]oxetane Derivatives

**DOI:** 10.3390/ma14195569

**Published:** 2021-09-25

**Authors:** Mateusz Korzec, Daiva Tavgeniene, Nizy Sara Samuel, Raminta Beresneviciute, Gintare Krucaite, Agnieszka Katarzyna Pająk, Sonia Kotowicz, Marharyta Vasylieva, Paweł Gnida, Jan Grzegorz Malecki, Saulius Grigalevicius, Ewa Schab-Balcerzak

**Affiliations:** 1 Institute of Chemistry, University of Silesia, 9 Szkolna Str., 40-006 Katowice, Poland; mateusz.korzec@us.edu.pl (M.K.); agpajak@us.edu.pl (A.K.P.); sonia.kotowicz@us.edu.pl (S.K.); jan.malecki@us.edu.pl (J.G.M.); 2 Department of Polymer Chemistry and Technology, Kaunas University of Technology, Radvilenu Plentas 19, LT50254 Kaunas, Lithuania; daiva.tavgeniene@ktu.lt (D.T.); nizysamuel@gmail.com (N.S.S.); raminta.beresneviciute@ktu.lt (R.B.); gintare.krucaite@ktu.lt (G.K.); 3 Centre of Polymer and Carbon Materials, Polish Academy of Sciences, 34 M. Curie-Sklodowska Str., 41-819 Zabrze, Poland; mvasylieva@cmpw-pan.edu.pl (M.V.); pgnida@cmpw-pan.edu.pl (P.G.)

**Keywords:** oxetane derivatives, carbazole materials, hole transporting materials

## Abstract

Novel oxetane-functionalized derivatives were synthesized to find the impact of carbazole substituents, such as 1-naphtyl, 9-ethylcarbazole and 4-(diphenylamino)phenyl, on their thermal, photophysical and electrochemical properties. Additionally, to obtain the optimized ground-state geometry and distribution of the frontier molecular orbital energy levels, density functional theory (DFT) calculations were used. Thermal investigations showed that the obtained compounds are highly thermally stable up to 360 °C, as molecular glasses with glass transition temperatures in the range of 142–165 °C. UV–Vis and photoluminescence studies were performed in solvents of differing in polarity, in the solid state as a thin film on glass substrate, and in powders, and were supported by DFT calculations. They emitted radiation both in solution and in film with photoluminescence quantum yield from 4% to 87%. Cyclic voltammetry measurements revealed that the materials undergo an oxidation process. Next, the synthesized molecules were tested as hole transporting materials (HTM) in perovskite solar cells with the structure FTO/b-TiO_2_/m-TiO_2_/perovskite/HTM/Au, and photovoltaic parameters were compared with the reference device without the oxetane derivatives.

## 1. Introduction

As a renewable energy source, solar energy is a very important way to solve environmental pollution and deficiency of current energy. Investigations into and development of efficient and cheap solar cells is a technological area where traditional silicon-based solar cells have been successfully commercialized regardless of some disadvantages. At this time, perovskite solar cells (PSCs) are regarded as one of the best alternatives to silicon-based photovoltaic (PV) devices. The intense heat required to purify silicon, the high amount of CO_2_ emitted during this process, as well as difficultly in deposition on flexible substrates can be listed as drawbacks of silicon PV. PSCs possess strong light absorption, high bipolar charge transporting possibility, long carrier diffusion length and lifetime as well as low exciton binding energy [1]. PSCs can be made with a low-temperature screen-printing process that is not only less energy-intensive but also less expensive. Moreover, it is believed that PSCs have lower carbon footprints and shorter energy payback periods than silicon [2]. Tremendous research efforts, which are devoted to the creation of high-power conversion efficiency PSCs, are highly related to the improved properties of newly developed hole transporting materials (HTM) [3]. Effective functions of the materials include extraction of photogenerated positive charges from a layer of perovskite, transportation of the charges to the metal electrode, and minimization of recombination losses at the TiO_2_/perovskite/HTM interface [4,5]. Considering the literature review, it can be concluded that spiro-OMeTAD is the best-known HTM. It should be noted that it is an expensive material due to its complicated synthesis, mediocre hole carrier mobility and negative impact on stability which significantly increase the production cost of PV devices and their stability [6]. Due to these reasons the synthesis and investigation of novel efficient HTM is one of the hottest research topics in the PSCs field [7,8,9,10]. Among the reported HTM, carbazole-based compounds have drawn much attention due to good charge-transport ability of the carbazole units and low-cost of synthesis of the derivatives, which have provided a wide range of HTM by simple synthesis methods [11,12,13]. 

In this work, we describe synthesis and characterization of new simple carbazole-based oxetanes with 1-naphthyl, 9-ethylcarbazolyl and 4-(diphenylamino)phenyl moieties. The oxetanyl group containing molecules can form hydrogen bonds in solid state and the materials demonstrate high melting temperature (T_m_) or high glass transition temperature (T_g_) as well as formation of stable and homogeneous thin films due to intermolecular interactions, which can be promising for applications in organic optoelectronics including photovoltaics. They were tested as HTM in PSCs. The presented systematical studies are focused on finding the effect of some structural elements on crucial properties of the synthesized compounds. In design of new efficient HTMs, the role of systematical photophysical and optoelectronic investigations, which can help understand how to optimize the charge extraction and recombination processes by fine-tuning of the molecular structures, is essential to develop PV devices with improved performance. The obtained results should allow modeling of the chemical structure to obtain a material with specific characteristics for PV application. Some of the derivatives demonstrated improved amorphous film-forming properties, and, applied as HTM, lead to improvement of PV device parameters when compared with a reference cell without HTM.

## 2. Experimental Methods

### 2.1. Materials

9*H*-Carbazole (**1**), 3,3-bis(chloromethyl)oxetane, 4-(diphenylamino)phenylboronic acid, 9-ethyl-9*H*-carbazole-3-boronic acid pinacol ester, naphthalene-1-boronic acid, bis(triphenylphosphine)palladium(II) dichloride (Pd(PPh_3_)_2_Cl_2_), K_2_CO_3_, KOH, KI, KIO_3_, tetra-n-butylammonium hydrogen sulfate (TBAHS), Na_2_SO_4_ and solvents were purchased from Sigma Aldrich (Merck, Darmstadt, Germany) and used as received. Materials used for prepared perovskites solar cells: fluorine-doped tin oxide coated glass slides (FTOs, 7 Ω/sq, Sigma-Aldrich), tetraethyl orthotitanate ((C_2_H_5_O)_4_Ti, Merck), hydrochloric acid (HCl, CHEMPUR), anhydrous ethanol (EtOH, POCH), paste Ti-Nanoxide T/SP (Solaronix), methylammonium iodide (MAI, Solaronix), isopropanol (IPA, POCH), chlorobenzene (C_6_H_5_Cl, POCH). Lead iodide (PbI_2_), anhydrous *N*,*N*-dimethylformamide (DMF), lithium bis(trifluoromethanesulfonyl)imide (Li-TFSI), 4-tert-butyl pyridine (TBP). All were purchased from Sigma-Aldrich. 3-Iodo-9*H*-carbazole (**2**) was synthesized according to the procedure described in the literature [14]. 

### 2.2. Synthesis of Oxetane Derivatives

3,3-Di[3-iodocarbazol-9-ylmethyl]oxetane (**3**) was synthesized and characterized according to the procedure described in the literature [15].

3,3-Di[3-(1-naphthyl)carbazol-9-ylmethyl]oxetane (**4**). 0.4 g (0.6 mmol) of 3,3-di[3-iodocarbazol-9-yl]methyloxetane (**3**), 0.26 g (1.5 mmol) of naphthalene-1-boronic acid, 0.02 g (0.03 mmol) of PdCl_2_(PPh_3_)_2_ and 0.17 g (3.0 mmol) of powdered KOH were stirred in 8 mL of THF containing degassed water (1 mL) at 80 °C under nitrogen for 1.5 h. After TLC control the reaction mixture was cooled and quenched by the addition of ice water. The product was extracted with chloroform. The combined extract was dried over anhydrous Na_2_SO_4_. The crude product was purified by silica gel column chromatography using the mixture of ethyl acetate and hexane (vol. ratio 1:7) as an eluent. Yield: 0.3 g of white crystals (75%). DSC: T_m_ = 250 °C. MS (APCI^+^, 20 V): 669.49 ([M+H], 100%). ^1^H NMR (400 MHz, CDCl_3_, δ:ppm): 8.21–8.04 (m, 4H, Ar), 7.93–7.79 (m, 6H, Ar), 7.56–7.28 (m, 16H, Ar), 7.26–7.20 (m, 2H, Ar), 4.75 (s, 4H, 2×OCH_2_), 4.72 (s, 4H, 2×NCH_2_). ^13^C NMR (100 MHz, CDCl_3_, δ:ppm): 141.90, 140.85, 140.65, 133.90, 132.52, 132.15, 128.38, 128.30, 127.41, 126.38, 126.28, 126.04, 125.76, 125.45, 123.46, 123.41, 122.09, 120.79, 119.98, 108.876, 108.44, 76.12, 50.79, 47.64. FT-IR (KBr), cm^−1^: 3051, 2954, 2923, 2853, 1941, 1733, 1623, 1600, 1572, 1535, 1490, 1465, 1394, 1330, 1290, 1264, 1226, 1156, 986, 968, 800, 776, 745.

3,3-Di[3-(9-ethylcarbazol-3-yl)carbazol-9-ylmethyl]oxetane (**5**). 0.4 g (0.6 mmol) of 3,3-di[3-iodocarbazol-9-yl]methyloxetane (**3**), 0.48 g (1.5 mmol) of 9-ethyl-9H-carbazole-3-boronic acid pinacol ester, 0.02 g (0.03 mmol) of PdCl_2_(PPh_3_)_2_ and 0.17 g (3.0 mmol) of powdered KOH were stirred in 8 mL of THF containing degassed water (1 mL) at 80 °C under nitrogen for 2 h. After TLC control the reaction mixture was cooled and quenched by the addition of ice water. The product was extracted with chloroform. The combined extract was dried over anhydrous Na_2_SO_4_. The crude product was purified by silica gel column chromatography using the mixture of ethyl acetate and hexane (vol. ratio 1:5) as an eluent. Yield: 0.25 g of white amorphous product (52%). MS (APCI^+^, 20 V): 803.58 ([M+H], 100%). ^1^H NMR (400 MHz, CDCl_3_, δ:ppm): 8.31–8.09 (m, 6H, Ar), 7.47–7.34 (m, 10H, Ar), 7.31–7.12 (m, 10H, Ar), 4.65 (s, 4H, 2×OCH_2_), 4.64 (s, 4H, 2×NCH_2_), 4.37–4.26 (m, 4H, 2×NCH_2_CH_3_), 1.38 (t, 6H, *J* = 6.8 Hz, 2×CH_3_). ^13^C NMR (100 MHz, CDCl_3_, δ:ppm): 141.90, 140.50, 140.44, 139.16, 134.44, 132.97, 126.24, 126.04, 125.78, 125.53, 123.95, 123.59, 123.57, 123.15, 120.77, 120.57, 119.80, 119.20, 119.09, 118.87, 108.99, 108.86, 108.73, 108.60, 76.07, 50.825, 47.52, 37.68, 13.90. FT-IR (KBr), cm^−1^: 3048, 2967, 2928, 2870, 1877, 1731, 1627, 1600, 1472, 1456, 1380, 1330, 1231, 1154, 1124, 1088, 1065, 1024, 969, 878, 798, 781, 745, 727.

3,3-Di[3-(4-(diphenylamino)phenyl)carbazol-9-ylmethyl]oxetane (**6**). 0.4 g (0.6 mmol) of 3,3-di[3-iodocarbazol-9-yl]methyloxetane (**3**), 0.43 g (1.5 mmol) of 4-(diphenylamino)phenylboronic acid, 0.02 g (0.03 mmol) of PdCl_2_(PPh_3_)_2_ and 0.17 g (3.0 mmol) of powdered KOH were stirred in 8 mL of THF containing degassed water (1 mL) at 80 °C under nitrogen for 1.5 h. After TLC control the reaction mixture was cooled and quenched by the addition of ice water. The product was extracted with chloroform. The combined extract was dried over anhydrous Na_2_SO_4_. The crude product was purified by silica gel column chromatography using the mixture of ethyl acetate and hexane (vol. ratio 1:7) as an eluent. Yield: 0.3 g of white amorphous product (56%). MS (APCI^+^, 20 V): 903.56 ([M+H], 100%). ^1^H NMR (400 MHz, CDCl_3_, δ:ppm): 8.24–8.06 (m, 6H, Ar), 7.63–7.55 (m, 2H, Ar), 7.51–7.33 (m, 7H, Ar), 7.29–7.19 (m, 12H, Ar), 7.14–7.03 (m, 12H, Ar), 6.99–6.90 (m, 5H, Ar), 4.65 (s, 4H, 2×OCH_2_), 4.61 (s, 4H, 2×NCH_2_). ^13^C NMR (100 MHz, CDCl_3_, δ:ppm): 147.82, 146.65, 141.86, 140.66, 135.95, 132.89, 129.30, 127.95, 126.32, 125.33, 124.41, 124.28, 123.87, 123.48, 122.807, 120.71, 119.90, 118.66, 108.96, 108.85, 75.99, 50.77, 47.49. FT-IR (KBr), cm^−1^: 3055, 3031, 2954, 2923, 2866, 1965, 1625, 1589, 1515, 1488, 1451, 1384, 1331, 1275, 1230, 1178, 1155, 1073, 1028, 969, 889, 803, 748, 696.

### 2.3. Instrumentation and Characterization Methods 

Instrumentation, characterization methods, description of DFT calculations, preparation of films and solar cells are presented in electronic supporting information (ESI). 

## 3. Results and Discussion

### 3.1. Synthesis and Structural Characterization 

**Figure 1:**
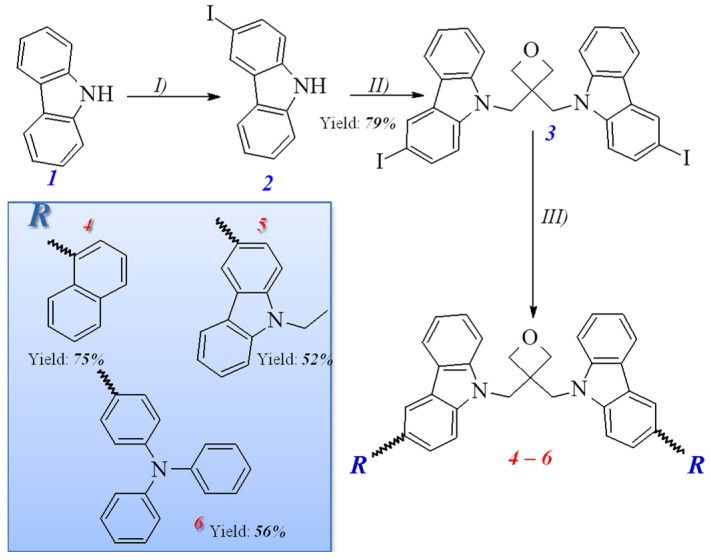
Synthesis of the carbazole-functionalized oxetane derivatives (**4**–**6**): (*I*) KI, KIO_3_, (*II*) KOH, K_2_CO_3_, TBAHS, 3,3-di(chloromethyl)oxetane, (*III*) Pd(PPh_3_)_2_Cl_2_, KOH, organoboron derivatives.

The new 3,3-di[3-(6-arylcarbazol-9-ylmethyl]oxetanes (**4**–**6**) were synthesized in the three synthesis steps as shown in Figure 1. 3-Iodo-9*H*-carbazole (**2**) was firstly prepared from commercially available 9*H*-carbazole (**1**) by using Tucker’s iodination procedure [14]. 3,3-Di(3-iodo-9-carbazolylmethyl)oxetane (**3**) was then synthesized as a key starting material by the reaction of 3,3-di(chloromethyl)oxetane with an excess of 3-iodo-9*H*-carbazole (**2**) in the presence of phase transfer catalyst (TBAHS) under basic conditions. Finally, the objective derivatives (**4**–**6)** were prepared by Suzuki reaction of the key starting diiodo-material **3** with an excess of 4-(diphenylamino)phenylboronic acid, 9-ethyl-9*H*-carbazole-3-boronic acid pinacol ester or naphthalene-1-boronic acid, correspondingly. The synthesized objective electroactive materials were identified by mass spectrometry, ^1^H and ^13^C NMR, and IR spectroscopy (cf. Figure S1). The data were observed to be in good agreement with the theoretical structures. IR spectra of synthesized compounds calculated using DFT method were well correlated with experimental ones (cf. see in ESI). 

The novel oxetane derivatives were soluble in common organic solvents. Transparent thin layers of these derivatives could be prepared on substrates by using spin coating from solutions technology.

### 3.2. Thermal Study 

**Figure 2:**
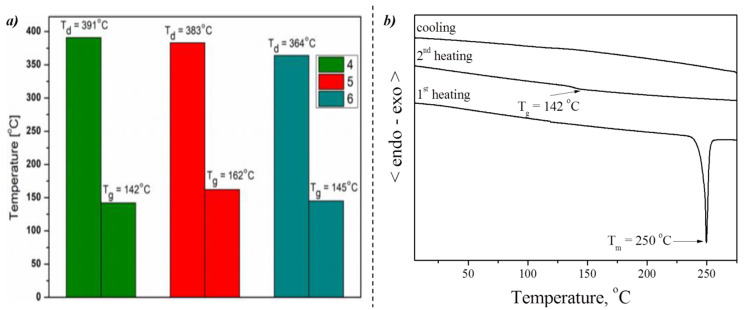
(**a**) Temperatures of the beginning of decomposition based on 5% weight loss (T_d_) from TGA curves and glass transition temperatures (T_g_) from second heating scan in DSC measurements and (**b**) DSC curves of the compound **4**.

Thermal properties of the synthesized oxetane-based derivatives (**4**–**6**) were established under a nitrogen atmosphere using thermogravimetric analysis (TGA) and differential scanning calorimetry (DSC). The results were summarized in Figure 2 (cf. Figures S2 and S3), and the representative DSC thermograms of **4** are presented in Figure 2b. 

Compounds (**4**–**6**) showed high thermal stability with the onset of thermal decomposition temperatures (T_d_) above 360 °C. The substitution of carbazole units with naphthalene structures (**4**) increased T_d_. In the DSC thermogram registered during the first heating of derivative **4**, an endothermic peak due to melting at 250 °C was observed (cf. Figure 2b).

When the melted sample was cooled down, it was transferred to the amorphous phase with T_g_ of 142 °C. Contrary to compounds with naphthalene units, compounds **5** and **6** were obtained after synthesis and purification directly as amorphous materials with T_g_ of 162 °C for **5** and 145 °C for **6**. The DSC thermograms of compounds **5** and **6** are demonstrated in Figure S3. in ESI. During the second heating scan, also only the glass-transition temperatures (T_g_) of the amorphous materials were observed, and on further heating, no peaks due to crystallization and melting appeared. The compounds showed high T_g_, which is advantageous considering the formation of a uniform and stable film during the PV cell operation at elevated temperature [10]. 

### 3.3. Electrochemical Measurements

**Figure 3:**
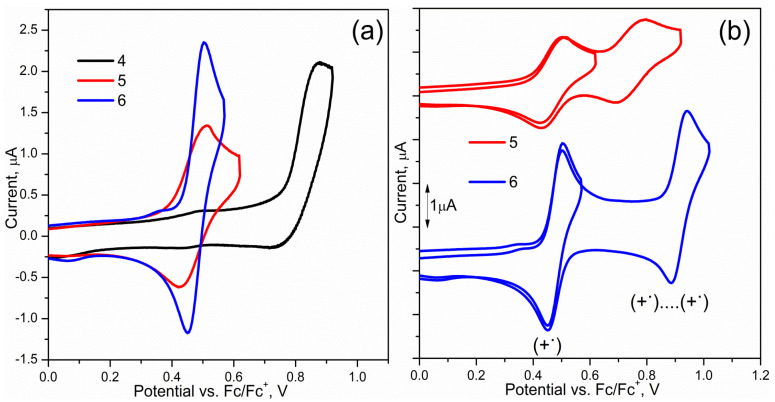
(**a**) CV curves of **4**, **5**, **6** in 0.1 M Bu_4_NPF_6_/CH_3_CN solutions. The measurements were performed with a platinum working electrode (Pt) and referenced against Fc/Fc^+^ couple, scanning rate 50 mV/s; (**b**) CV curves of **4** and **5** solution (1st and 2nd oxidation peaks).

The synthesized carbazole-functionalized oxetane derivatives were examined by cyclic voltammetry (CV) in acetonitrile (CH_3_CN). The compounds underwent an oxidation process (cf. Figure 3 and Figure S4). HOMO energy level, assuming that the ionization potentials (IP) of ferrocene equals −5.1 [15], was calculated based on the electrochemical oxidation onset potential (cf. Table 1). 

The reduction process of compounds (**4**–**6**) was not observed from 0 to −2.4 V. In this case, LUMO levels were estimated using the energy gap from the UV–vis spectrum and the optical energy gaps (E_g_^OPT^) are in the range from 3.25 eV (**5**) to 3.45 eV (**4**). The oxidation process of the molecule with naphthalene substituent (**4**) was characterized as irreversible behavior (cf. Figure 3a), and its oxidation potential (0.76 V) is higher than others (**5** and **6**). 

**Figure 4:**
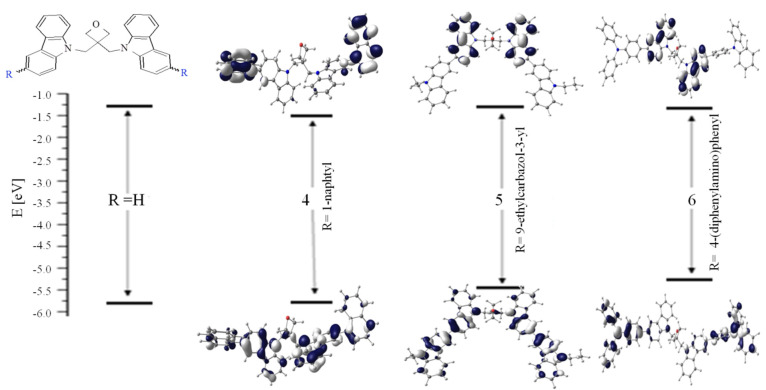
HOMO and LUMO contours and orbital energy changes associated with the introduction of substituents to 3,3-di[3-arylcarbazol-9-ylmethyl]oxetane.

The oxidation process of derivatives functionalized with 9-ethylcarbazolyl (**5**) and 4-(diphenylamino)phen (**6**) was characterized as a quasi-reversible process, with onset oxidation potentials at 0.43 V (**5**) and 0.38 V (**6**), respectively (cf. Table S1). These values correspond with the literature [16,17,18]. The first oxidation process is assigned to an electron extraction from HOMO. During scanning to a higher potential a second oxidation peak with onsets at 0.68 V (**5**) and 0.86 V (**6**) (in the range from 0 V to 1.6 V vs. Fc/Fc^+^) were observed (cf. Figure 3b). The ratio of the first oxidation peak to the second is 1:1 in both cases. First, the oxidation potential formed a radical cation (+^·^); second, the oxidation potential formed a di-cation (+^·^….+^·^), or donor groups were oxidized in sequence. Based on the calculated composition of the selected molecular orbitals (cf. Table S2, Figure 4) it can be concluded that HOMO in **4** is mainly localized on the carbazolyl moiety, in **5** this includes carbazole together with the *N*-ethylcarbazolyl substituent, while in the case of **6**, triphenylamine plays a dominant role in the formation of the HOMO level. During several cycles of the oxidation process, oxetane derivatives did not form any product on the surface of an electrode (cf. Figure S4). Comparing the energies of frontier molecular orbitals determined on the basis of electrochemical data with DFT calculated values, it can be noticed that the calculated HOMO energies do not differ much from the experimental values (cf. Table 2). 

In the case of LUMO the differences are greatly pronounced as can be expected, but the same tendency, that is, the lowering of LUMO in the following order: **6**, **5**, **4** was seen. Based on energy changes in the frontier orbitals energy (Figure 4), it can be concluded that the strongest electron-donating property is shown by 4-(diphenylamino)phenyl substituent, and the lowest by 1-naphtyl. Considering the HOMO energy level of the investigated compounds it seems that oxetane with triphenylamine unit (**6**) is the most thermodynamically capable of extracting holes from perovskite, with a valence band edge at around 5.4 eV [19]. On the other hand, the LUMO levels of oxetane derivatives around −2.3 eV can effectively block electron transport from perovskite to the Au electrode, preventing carrier recombination at the anode [20]. Thus, the LUMO levels are higher than the conduction band of perovskite, making them good electron blocking materials.

### 3.4. Photophysical Investigations 

UV–Vis absorption and photoluminescence (PL) spectra of synthesized molecules were measured in the solvents with various polarities (chloroform < chlorobenzene < dichloromethane < methanol < acetonitrile), in the solid state as a thin film on glass substrate and as powders. The measurements were performed in the same conditions and the same compound concentrations. The obtained spectroscopic data are listed in Table 3, whereas the UV–Vis and PL spectra are depicted in Figure 4 and Figures S5–S8 in ESI.

Investigated compounds absorbed in the range of 250 nm to 380 nm with one dominating band. They show optical energy band gaps (E_g_^OPT^) in the range of 3.25–3.45 eV. Low absorption and a rather wide band gap of the hole transporting material is beneficial in reducing the optical losses [21]. Considering the absorption, excitation, and emission spectra of the functionalized oxetane derivatives in various solvents (cf. Figure 4a and Figure S5), the effect of the carbazole substituents chemical structure is pronounced. The molecules with 1-naphtyl (**4**) and 9-ethylcarbazol-3-yl (**5**) substituents exhibited absorption bands with maximum (λ_max_) at about 273–300 nm, while λ_max_ of the compound with a 4-(diphenylamino)phenyl units (**6**) was shifted to ~325 nm (cf. Table 3, Figure S5). In the UV–Vis spectra of compound **4** the λ_max_ located at 293–297 nm was dominating, except in acetonitrile where λ_max_ = 273 nm. It should be noted that the observed changes are visible only for this derivative and only in acetonitrile (cf. Figure S6a). Therefore, it may indicate a strong interaction between the compound with the naphthalene substituent and the solvents such as acetonitrile [22,23]. The calculated absorption spectra in acetonitrile, dichloromethane, chlorobenzene and chloroform solvents exhibit differences in the band maxima only in the case of compound **4**; in **5** and **6** the differences are negligible (cf. Figure S6a). A small solvatochromic effect visible in the case of **4** and practically no changes in the band maxima on absorption spectra of **5** and **6** in solvents with different polarity is likely associated with relatively small changes in the dipole moments between the ground and first excited states. In the case of compound **4**, the change in the dipole moment between states S_0_ and S_1_ is 0.7 D, and for compounds **5** and **6**, these values are 0.03 and 0.25 D, respectively (cf. Table S3). Moreover, only in the case of compound **4**, the influence of the solvent polarity on the emission quantum yield (Φ_CH2Cl2_ = 31.8%, Φ_CH3CN_ = 87%, cf. Table 3) is noticeable, in the other two cases the changes in the quantum yield between methylene chloride and acetonitrile solutions are negligible. According to the TD-DF calculation, the excitation electronic transitions (λ_ex_ = 300 nm (**4**), 306 nm (**5**) and 330 nm (**6**) in CH_2_Cl_2_) correspond to the H-2/H-1/HOMO→LUMO/L+1/L+2/L+3 type (cf. Table S4). Taking into account the nature of the energy levels involved in these transitions, it can be seen that in the case of **4** the excitation has intramolecular charge transfer nature (**π**_carbazole_ → **π**_R_^*^) and in **5** and **6** the excitation has mixed intramolecular charge transfer/locally-excited (ICT/LE) character (**π**_carbazole_ + **π**_R_ → **π**_R_^*^). The absorption spectra of the compounds in the film compared to the solution spectra showed bathochromic shifts due to molecular packing in the solid state.

The emission and excitation spectra were registered and for the most intense PL band in the selected media the PL quantum yield (Φ_PL_) was measured (cf. Table 3). The functionalized oxetane derivatives emitted in the near-ultraviolet and the violet spectral range with the main maximum emission band (λ_em_) located in the range of 373–419 nm. However, a weak second emission band with the λ_em_ at lower energies was also observed. This behavior was also noticed and presented in a previous paper [24], but in that case, the presence of the low-energy emission band was due to the presence of copper ions. In order to explain the presence of the second PL band, DFT calculations were performed. The first singlet and triplet excited states were optimized and, the calculated energy differences between T_1_ and S_0_ states corresponded to the long-wave band on the emission spectra (cf. Table S3). So, the presence of this low-energy band indicates the processes of internal conversion of the excited state, and the compounds exhibit phosphorescence at room temperature.

**Figure 5:**
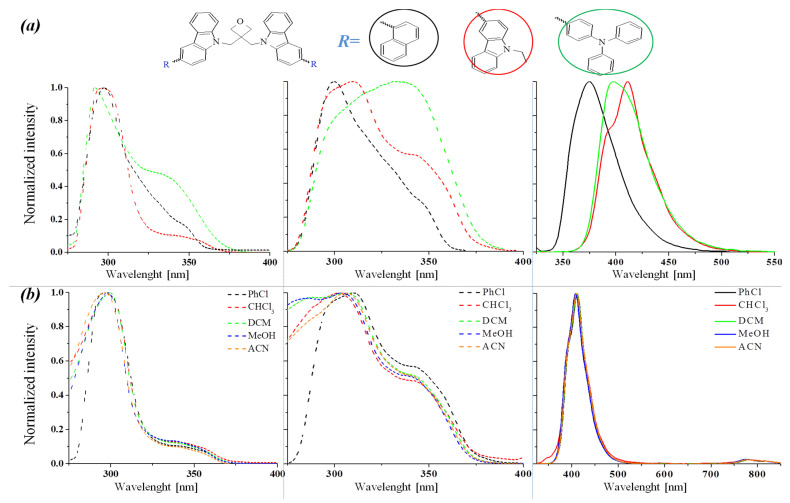
Imposed UV–Vis and excitation spectra (*dotted line*) as well as PL spectra (*solid line*) for: (**a**) compounds (**4**, **5**, **6**) in chlorobenzene (PhCl); (**b**) compound ***5*** in various solvents such as: chloroform (CHCl_3_), chlorobenzene (PhCl), dichloromethane (CH_2_Cl_2_), methanol (CH_3_OH) and acetonitrile (CH_3_CN).

An influence of solvent polarity on the positions of λ_max_ and λ_em_ was not seen, thus the solvatochromism effect was not observed (cf. Figure 5b and Figure S6). However, the influence of polarity on the quantum yield was noticeable (cf. Table 3). In the chloroform solution weak PL was noted, while in dichloromethane or acetonitrile PL intensity significantly increased. The quantum yield in chloroform ranged from 2.1% to 19%, while in acetonitrile quantum yield ranged from 25.5% to 87%. Moreover, the PL intensity dependencies on the dielectric constant of the solvents were also visible in the recorded spectra at different excitation wavelengths (cf. Figure S7). The influence of polarity on the quantum yield of oxetane derivatives was reported in other works [25]. The slightly bathochromic shift of the λ_em_ in the films and powders relative to the solutions were observed (cf. Table 3).

**Figure 6:**
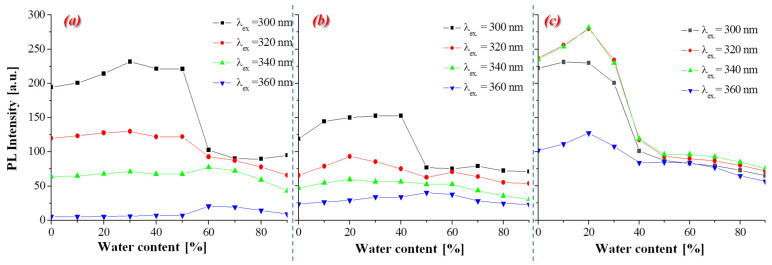
Changes in the PL intensity versus the water content (***f_w_***) in a binary mixture of CH_3_CN/H_2_O with an increasing water content (***f_w_*** = 0%, 10%, 20%, 30%, 40%, 50%, 60%, 70%, 80%, 90%, *v*/*v*) at different excitation wavelengths (λ_ex_.: 300, 320, 340, 360 nm) for: (**a**) ***4***, (**b**) ***5***, (**c**) ***6*** (cf. Figure S12).

Additionally, the effect of the aggregation phenomenon on optical properties of functionalized oxetane derivatives in a binary mixture of CH_3_CN/H_2_O with increasing water content (***f_w_*** = 0%, 10%, 20%, 30%, 40%, 50%, 60%, 70%, 80%, 90%, *v*/*v*) was investigated. The obtained results are shown in Figure 6 and Figure S9 in ESI. Measurements were carried out using four different excitation wavelengths (λ_ex_): 300, 320, 340, 360 nm. All compounds showed a trend towards aggregation-caused quenching (ACQ), where increasing the water content in the CH_3_CN/H_2_O system above 40% (in the case of **6** above 30%) caused a reduction in the emission intensity [26,27,28] (cf. Figure 6). Moreover, it can be seen that in the aggregation state (from 50% to 90% *v*/*v* of water content), compound **6** with 4-(diphenylamino)phenyl units have shown a similar emission intensity for different λ_ex_ in contrast to molecules with 1-naphthyl (**4**) and 9-ethylcarbazolyl (**5**) substituents. The aggregation investigations have demonstrated that different water content in the CH_3_CN/H_2_O system did not affect the λ_em_ (cf. Figure S9). 

### 3.5. Photovoltaic Characterization

**Figure 7:**
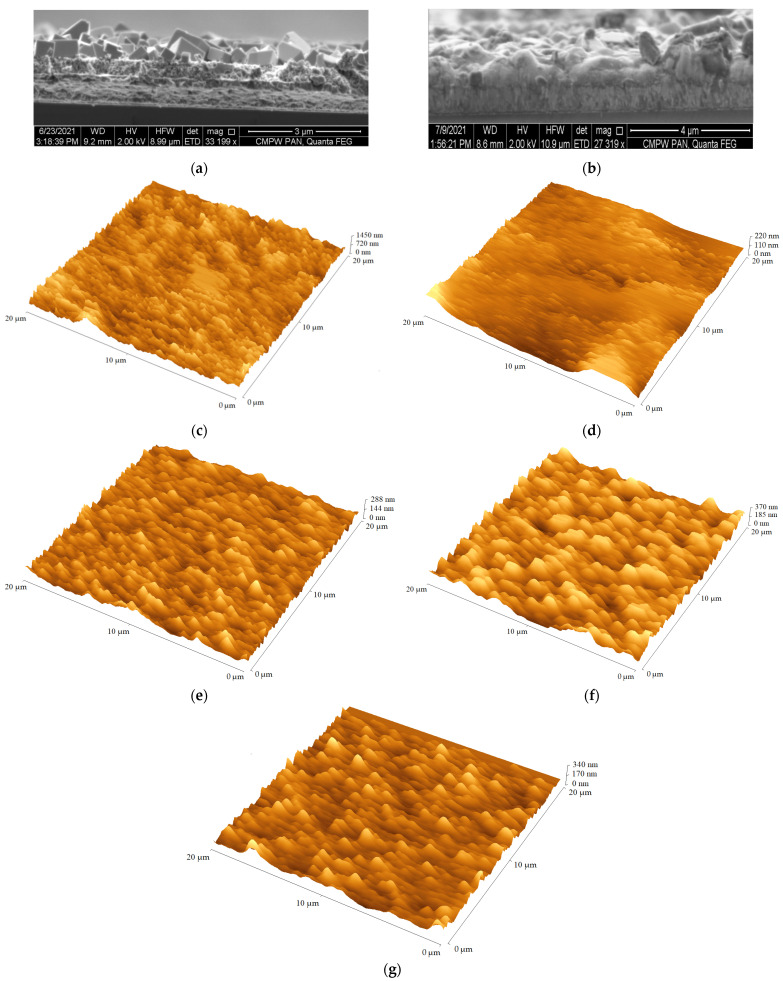
Cross-section SEM image of the reference (**a**) FTO/b-TiO_2_/m-TiO_2_/perovskite and with HTM (**b**) FTO/b-TiO_2_/m-TiO_2_/perovskite/HTM (on the right) perovskite solar cells and the AFM micrograms of (**c**) FTO/b-TiO_2_/m-TiO_2_, (**d**) FTO/b-TiO_2_/m-TiO_2_/perovskite, (**e**) FTO/b-TiO_2_/m-TiO_2_/perovskite/**4** (with 17.50 μL Li-TFSI), (**f**) FTO/b-TiO_2_/m-TiO_2_/perovskite/**6** (with 35.00 μL Li-TFSI) and (**g**) FTO/b-TiO_2_/m-TiO_2_/perovskite/**5** (with 8.75 μL Li-TFSI).

The synthesized oxetane derivatives were tested as hole transporting materials in perovskite solar cells to find the effect of carbazole substituent structure on device performance. Prior to solar cell fabrication, the surface quality of the prepared layers was evaluated using an atomic force microscope (AFM), and the root-mean-square (RMS) parameter was determined. For this purpose, RMS values were determined for the following layers: FTO/b-TiO_2_/m-TiO_2_, FTO/b-TiO_2_/m-TiO_2_/perovskite, and the last one was FTO/b-TiO_2_/m-TiO_2_/perovskite/HTM and the AFM micrograms are given in Figure 7b–g. The perovskite layers were obtained by the two-step method and the details are given in ESI. According to the predictions, the smoothest layer was FTO/b-TiO_2_/m-TiO_2_ with RMS values in the range of 20–25 nm. After applying perovskite, the layers’ roughness increased significantly to values of 170–180 nm. Next, it was found that the oxetane derivatives fully covered the perovskite surface and reduced its surface fluctuation. The RMS value was reduced to 45, 40 and 55 nm for compounds **4**, **5**, and **6,** respectively. A beneficial effect of perovskite surface smoothing due to the presence of oxetane derivatives on the short-circuit current density (J_sc_) and open-circuit voltage (V_oc_) is expected.

Non-encapsulated devices with the structure FTO/b-TiO_2_/m-TiO_2_/perovskite/HTM/Au were fabricated. Additionally, the reference solar cell without oxetane derivatives was also prepared (cf. Figure 7a). The details of the PSCs’ construction are described in the ESI. The cross-sectional scanning electron microscopy (SEM) images of the reference device and devices with oxetane derivatives are presented in Figure 7a. The HTM layer was doped with the most common additive of lithium bis(trifluoromethanesulfonyl)imide (Li-TFSI). Different doping volumes of Li^+^: 8.75, 17.50 and 35.00 μL were applied to find the optimal amount of dopant to give the best PV performance. The calculated photovoltaic parameters (J_sc_—short-circuit current, V_oc_—open-circuit voltage, FF—fill factor, and PCE—power conversion efficiency) based on current–voltage (I–V) characteristics of the prepared devices are collected in Table 4. The I–V characteristics were registered in a forward and backward scan (measurements were conducted in the Standard Test Conditions) and the I–V characteristics for champion devices are presented in ESI as Figure S10. 

Taking into account the I–V characteristics registered in a forward and backward scan, the hysteresis, which often appears in PCSs, was seen [29]. Hysteresis had unfavorable impacts on photovoltaic performance of PSCs [30,31]. The PCE was lower in the range of 0.07–0.72% than that of the forward scan, which is a rather significant value considering the low value of PCE exhibited by fabricated devices. The obtained results showed the effect of both structure of carbazole substituent and dopant concentration on PV parameters. The application of a molecule with 9-ethylcarbazolyl units (**5**) increased V_oc_ but reduced the J_sc_ and finally resulted in lower PCE compared to the reference device. Higher PCE values were obtained for the cells with **4** and **6** compounds in all doping concentrations. However, the amount of Li-TFSI, which prevents the highest conversion efficiency, was different for device with **4** and **6**. The optimal V_Li-TFSI_ for PSC with oxetane bearing naphthalene (**4**) and triphenylamine structures (**6**) was 17.50 and 35.00 μL, respectively. Such doping concentrations raise the PCE mainly due to an increase in J_sc_. The highest V_oc_ above 600 mV was observed for the device with compound **6** relative to the others, which can be explained by their differences in HOMO energy levels [10]. Moreover, cells based on oxetane substituted with 4-(diphenylamino)phenyl moieties (**6**) showed the highest PCE of 2.94%. 

## 4. Conclusions

The carbazole functionalized oxetanes substituted with the 1-naphthyl, 9-ethylcarbazolyl and 4-(diphenylamino)phenyl units were synthesized and investigated. Considering the impact of carbazole substituent structures it was found that:

- molecules with 9-ethylcarbazol-3-yl (**5**) and 4-(diphenylamino)phenyl (**6**) substituents were obtained as amorphous materials. The presence of 9-ethylcarbazolyl (**5**) units raised the glass transition temperature by about 20 °C when compared with the others;
- 1-naphthyl (**4**) units increased PL quantum yields both in solution and in film (87% in solutions and 9% in film), hypsochromically shifted λ_em_, lowered the HOMO energy level to −5.86 eV, broadened the energy gap to 3.45 eV, and raised J_sc_ and V_oc_ of the device compared to the reference cell,
- 9-ethylcarbazolyl (**5**) moieties reduced the PL quantum yield, narrowed the bandgap to 3.25 eV and significantly reduced J_sc_ which negatively affected the PCE of the device,
- the 4-(diphenylamino)phenyl (**6**) substituents raised the HOMO energy level to −5.48 eV, which has a beneficial impact on V_oc_ of device.

It can be concluded that utilization of oxetane derivatives with 4-(diphenylamino)phenyl units in perovskite cell led to a 2.5 times increase in PCE values in relation to the reference device, likely due to its HOMO energy level best matching to perovskite. Further, chemical modification of this compound seems to be promising for improving its properties toward efficient hole transporting materials. 

## Figures and Tables

**Table 1:** Electrochemical data for samples **4**, **5**, **6** recorded in 0.1 M Bu_4_NPF_6_/CH_3_CN solution.

Code	E_ox_^onset^ [V]	HOMO [eV]	HOMO^DFT^ [eV]	E_g_^OPT^ [eV]	LUMO [eV]	LUMO^DFT^ [eV]
**4**	0.76	−5.86	−5.78	3.45	−2.41	−1.51
**5**	0.43	−5.53	−5.49	3.25	−2.28	−1.37
**6**	0.38	−5.48	−5.26	3.32	−2.16	−1.33

HOMO = −5.1 − E_ox_^onset^, E_g_^OPT^ = 1240/λ, LUMO = E_g_^OPT^ − HOMO, Pt, v = 50 mV/s with c = 10^−3^ mol/dm^3^ and 0.1 M Bu_4_NPF_6_/CH_3_CN.

**Table 2:** DTF results of dipole moments and HOMO, LUMO orbitals energy.

Code	Dipole Moment	HOMO	LUMO
**4**	2.62	−5.78	−1.51
**5**	3.89	−5.26	−1.33
**6**	4.86	−5.49	−1.37

**Table 3:** The UV–Vis and PL spectroscopic parameters of the synthesized oxetane derivatives.

Code	Medium ^a^	λ_max_ [nm] (ε·10^4^) ^b^	λ_ex_ [nm]	PL		
				λ_em_ [nm]	Stokes Shift ^c^ [cm^−1^]	Φ_PL_ (%)
**4**	CHCl_3_	272 **^sh^**, 296(13.4), 345 **^sh^**	263 ^sh^, **300**, 350 **^sh^**	360 **^sh^**, **373**, 716, 746	6974	19
	*PhCl*	297(5.0), 345 ^sh^	**300**, 350 **^sh^**	360 **^sh^**, **373**, 714, 746	6860	-
	*CH_2_Cl_2_*	296(7.3), 345 ^sh^	262, **300**, 345 **^sh^**	360 **^sh^**, **374**, 714, 746	7046	31.9
	*CH_3_OH*	293(10.4), 345 ^sh^	262 **^sh^**, **300**, 345 **^sh^**	360 **^sh^**, **374**, 714, 746	7392	-
	*CH_3_CN*	273(14.1), 345 ^sh^	262, **300**, 315 **^sh^**, 345 **^sh^**	360 **^sh^**, **380**, 719, 751	10,314	87.0
	*Film*	**351**	**349**	**381**	2243	9.0
	*Powder*	-	260, **355**	372 **^sh^**, **397**	-	-
**5**	*CHCl_3_*	300(11.8), 345 ^sh^	286 **^sh^**, **302**, 345 **^sh^**	390 **^sh^**, **410**, 773, 820	8943	2.1
	*PhCl*	297(11.9), 345 ^sh^	300 **^sh^**, **310**, 343 **^sh^**	390 **^sh^**, **410**, 780, 820	9278	-
	*CH_2_Cl_2_*	300(11.9), 345 ^sh^	265, 286 **^sh^**, **306**, 342 **^sh^**	390 **^sh^**, **410**, 778, 815	8943	23.6
	*CH_3_OH*	300(14.4), 345 ^sh^	264, 284 **^sh^**, **303**, 343 **^sh^**	390 **^sh^**, **408**, 773, 815	8943	-
	*CH_3_CN*	295(12.6), 345 ^sh^	262, **305**, 342 **^sh^**	390 **^sh^**, **409**, 776, 820	9508	25.5
	*Film*	311 ^sh^, **360**	**305**, 348 **^sh^**,359 ^sh^	397 **^sh^**, **419**	3911	4.2
	*Powder*	**-**	261, 299 **^sh^**, 334 **^sh^**, **369**	**418**	-	-
**6**	*CHCl_3_*	277 ^sh^, 326(7.5)	**335**	**400**, 775, 802	11,101	4.3
	*PhCl*	290(11.6), 338 ^sh^	**335**	**400**, 776, 805	9483	-
	*CH_2_Cl_2_*	273 ^sh^, 328(6.6)	274, 300 **^sh^**, 315 **^sh^**, **330**	**406**, 782, 797	5857	43.0
	*CH_3_OH*	273 ^sh^, 323(12.2)	300 **^sh^**, 315 ^sh^, **342**	**406**, 771, 793	6329	-
	*CH_3_CN*	275 ^sh^, 302 ^sh^, 323(8.4)	300 **^sh^**, 315 **^sh^**, **330**	**409**, 780, 808	6510	42.8
	*Film*	**350**	**308**, 330 **^sh^**	**403**	3757	7.2
	*Powder*	-	**384**, 398 **^sh^**	**414**	-	-

^a^ Dielectric constant of solvents: chloroform (CHCl_3_, *ε = 4.89*), chlorobenzene (PhCl, *ε = 5.62*), dichloromethane (CH_2_Cl_2_, *ε = 8.93*), methanol (CH_3_OH, *ε = 32.66*) and acetonitrile (CH_3_CN, *ε = 35.94*). **^b^** Molar absorption coefficient (dm^3^/mol·cm). **^c^** Stokes shifts calculated according to the equation Δν = (1/λ_max_ − 1/λ_em_)·10^7^ [cm^−1^]. **^sh^**—shoulder. Bold data indicates the most intense band. Concentrations of the solutions were equal 10^−5^ mol/dm^3^.

**Table 4:** Photovoltaic properties of fabricated perovskite solar cells.

Device Structure	V_Li-TFSI_	I_sc_	J_sc_	V_oc_	FF	PCE
	[μL]	[mA]	[mA/cm^2^]	[mV]	[-]	[%]
**FTO/b-TiO_2_/m-TiO_2_/perovskite/Au**	–					
**Champion**		2.15	8.60	393.9	0.28	1.11
**Forward**		2.18 ± 0.04	8.71 ± 0.16	362.1 ± 72.6	0.28 ± 0.00	1.01 ± 0.24
**Backward**		1.89 ± 0.67	7.58 ± 2.67	355.4 ± 173.5	0.23 ± 0.04	0.72 ± 0.48
**FTO/b-TiO_2_/m-TiO_2_/perovskite/4/Au**	8.75					
**Champion**		2.79	11.14	514.3	0.26	1.53
**Forward**		2.65 ± 0.30	10.59 ± 1.21	509.6 ± 6.2	0.27 ± 0.01	1.46 ± 0.09
**Backward**		2.20 ± 0.07	8.81 ± 0.30	475.9 ± 22.4	0.25 ± 0.01	1.07 ± 0.06
**FTO/b-TiO_2_/m-TiO_2_/perovskite/4/Au**	17.50					
**Champion**		3.28	13.13	541.6	0.26	1.86
**Forward**		3.11 ± 0.36	12.43 ± 1.43	527.4 ± 16.1	0.25 ± 0.02	1.65 ± 0.33
**Backward**		2.52 ± 0.07	10.10 ± 0.26	522.9 ± 31.8	0.23 ± 0.01	1.25 ± 0.07
**FTO/b-TiO_2_/m-TiO_2_/perovskite/4/Au**	35.00					
**Champion**		3.18	12.70	544.8	0.25	1.77
**Forward**		3.04 ± 0.26	12.14 ± 1.06	561.4 ± 32.70	0.25 ± 0.01	1.70 ± 0.11
**Backward**		2.32 ± 0.23	9.29 ± 0.90	514.0 ± 12.44	0.23 ± 0.01	1.10 ± 0.09
**FTO/b-TiO_2_/m-TiO_2_/perovskite/5/Au**	8.75					
**Champion**		1.36	5.45	450.8	0.26	0.73
**Forward**		1.37 ± 0.03	5.50 ± 0.11	446.7 ± 10.2	0.26 ± 0.01	0.72 ± 0.04
**Backward**		1.13 ± 0.31	4.51 ± 1.24	428.4 ± 15.5	0.25 ± 0.01	0.54 ± 0.11
**FTO/b-TiO_2_/m-TiO_2_/perovskite/5/Au**	17.50					
**Champion**		1.14	4.55	464.3	0.25	0.60
**Forward**		1.02 ± 0.56	4.09 ± 2.25	472.5 ± 14.6	0.27 ± 0.16	0.57 ± 0.09
**Backward**		0.59 ± 0.06	2.38 ± 0.24	475.2 ± 9.1	0.22 ± 0.01	0.28 ± 0.04
**FTO/b-TiO_2_/m-TiO_2_/perovskite/5/Au**	35.00					
**Champion**		1.19	4.74	500.9	0.25	0.67
**Forward**		1.05 ± 0.47	4.20 ± 1.87	487.0 ± 8.6	0.24 ± 0.01	0.57 ± 0.29
**Backward**		0.71 ± 0.25	2.85 ± 0.98	482.6 ± 10.5	0.23 ± 0.00	0.37 ± 0.14
**FTO/b-TiO_2_/m-TiO_2_/perovskite/6/Au**	8.75					
**Champion**		3.36	13.43	379.8	0.26	1.36
**Forward**		3.35 ± 0.10	13.40 ± 0.41	382.2 ± 16.3	0.26 ± 0.00	1.35 ± 0.01
**Backward**		2.87 ± 0.14	11.48 ± 0.58	349.3 ± 0.92	0.21 ± 0.00	0.85 ± 0.03
**FTO/b-TiO_2_/m-TiO_2_/perovskite/6/Au**	17.50					
**Champion**		2.95	11.80	628.8	0.25	1.88
**Forward**		2.55 ± 0.46	10.22 ± 1.83	661.8 ± 33.86	0.25 ± 0.00	1.72 ± 0.27
**Backward**		2.36 ± 0.10	9.43 ± 0.39	714.7 ± 27.63	0.26 ± 0.01	1.79 ± 0.11
**FTO/b-TiO_2_/m-TiO_2_/perovskite/6/Au**	35.00					
**Champion**		4.26	17.03	757.5	0.23	2.94
**Forward**		4.25 ± 0.04	17.01 ± 0.16	754.7 ± 3.37	0.22 ± 0.01	2.91 ± 0.04
**Backward**		4.32 ± 0.21	17.26 ± 0.84	752.0 ± 12.09	0.21 ± 0.00	2.74 ± 0.18

**V_Li-TFSI_**—volume of additive of solution Li-TFSI; – without HTM; **Champion**—device with the best PCE parameters.

## Data Availability

The data presented in this study are available on request from the corresponding author.

## Supplementary Materials

The following are available online at https://www.mdpi.com/article/10.3390/ma14195569/s1, Figure Figure S1. Mass spectra, ^1^H NMR and ^13^C NMR spectra of compounds 4, 5 and 6, Figure S2. TGA curves of compounds: (a) 4, (b) 5, (c) 6. Figure S3. DSC curves of the compounds: (a) 5, (b) 6. Figure S4. Additional scans for compounds 4, 5 and 6. Figure S5. Imposed UV–Vis and excitation spectra, respectively (*dotted line*) as well as PL spectra (*solid line*) of compounds (4, 5 and 6) in various solvents: (a) chloroform (CHCl_3_), (b) dichloromethane (CH_2_Cl_2_), (c) methanol (CH_3_OH), d) acetonitrile (CH_3_CN). Figure S6. Imposed UV–Vis and excitation spectra, respectively (dotted line) as well as PL spectra (solid line) in various solvents for compound: (a) 4, (b) 6. Figure S6a. Calculated electronic absorption spectra of compounds 4, 5 and 6 in selected solvents. Figure S7. 3D spectra for the analyzed compounds (4, 5, 6) in the excitation range from 230 to 400 nm and the collected emissions in the range from 300 to 550 nm in three solvents: chloroform (CHCl_3_), dichloromethane (CH_2_Cl_2_) and acetonitryl (CH_3_CN). Figure S8. The excitation (dotted line) PL spectra (solid line) for compounds in (a) powder, (b) film. Figure S9. Experimental and calculated IR spectra of 4, 5 and 6 compounds. Figure S10. The optimized geometries of compounds 4, 5 and 6. Figure S10a. Optimized geometries of ground (green), S1 (red) and T1 (blue) states of compounds 4, 5 and 6. (Hydrogen atoms were omitted for clarity). Figure S11. DOS spectra of the investigated compounds. Figure S12. The photoluminescence (PL) properties of tested compounds (*10 μM concentration*) in a binary mixture of CH_3_CN/H_2_O with an increasing water content (*fw* = 0, 10, 20, 30, 40, 50, 60, 70, 80, 90%, *v*/*v*) at different excitation wavelengths (_ex._: 300, 320, 340, 360 nm) for: (a) *4*, (b) *5*, (c) *6*. Figure S13. I–V characteristics of the champion devices. Table S1. Number of electrons during oxidation process. Table S2. Composition of the selected molecular orbitals. Table S3. Energy differences [nm] between ground and S_1_, T_1_ excited states calculated in CH_2_Cl_2_ solvent (the maxima of emission wavelength are given in parenthesis) and calculated dipole moments. Table S4. The calculated electronic transitions corresponding to excitation wavelength in CH_2_Cl_2_ solution.
