# Supplementary material for: Synthesis and Thermal, Photophysical, Electrochemical Properties of 3,3-di[3-Arylcarbazol-9-ylmethyl]oxetane Derivatives
Source: Materials (Basel). 2021 Sep 25;14(19):5569. doi: 10.3390/ma14195569 (PMC8509391; doi:10.3390/ma14195569)
Supplement: Supplementary file 1 [file materials-14-05569-s001.zip › materials-1330496-supplementary.pdf]

materials

*Supplementary Materials*

# Synthesis and Thermal, Photophysical, Electrochemical Properties of 3,3-di[3-Arylcarbazol-9-ylmethyl]oxetane Derivatives

**Mateusz Korzec, Daiva Tavgeniene Nizy Sara Samuel, Raminta Beresneviciute, Gintare Krucaite, Agnieszka Katarzyna Pająk, Sonia Kotowicz, Marharyta Vasylieva, Paweł Gnida, Jan Grzegorz Malecki, Saulius Grigalevicius* and Ewa Schab-Balcerzak ***

## Instrumentation and characterization methods

$^{1}H$ NMR (400 MHz) and $^{13}C$ NMR (100 MHz) spectra were recorded by using Varian Unity Inova equipment. Mass spectra (MS) were obtained on Waters ZQ 2000 apparatus. IR spectra were recorded by using a Vertex 70 Bruker spectrophotometer.

Differential scanning calorimetry (DSC) measurements were performed by using a Bruker Reflex II thermos-system. The DSC curves have been recorded in a nitrogen atmosphere at a heating rate of 10° C/min. Thermogravimetric analysis tests (TGA) were carried out on TGAQ50 equipment. The TGA measurements were done at a heating rate of 10° C/min in a nitrogen atmosphere.

The electrochemical three-component cell comprised of the platinum electrode with a 1 mm diameter of Pt (platinum electrode) as a working electrode, an Ag|$Ag^{+}$ electrode as a pseudoreference electrode and a platinum coil as an auxiliary electrode. Electrochemical studies were undertaken in 0.1 M solutions of $Bu_4PF_6$, 99% (Sigma Aldrich) in $CH_3CN$. Measurements were conducted at room temperature at a potential rate of 50 mV/s and were calibrated against a ferrocene/ferrocenium redox couple ($Fc/Fc^{+}$). Electrochemical measurements were conducted in 1.0 mM concentrations of all compounds for all cyclic voltammetry (CV) measurements.

UV–Vis absorption spectra were measured using a Thermo Scientific Evolution 200 series UV–Visible Spectrophotometer for compound concentration of $10^{-5}$ mol/dm$^{3}$in 1cm quartz cell. Photoluminescence (PL) spectra in solution were measured using a Varian Cary Eclipse Spectrometer. Thin films and powders were measured on a Edinburgh Instruments FLS-980 fluorescence spectrophotometer equipped with a 450 W Xe lamp and highgain photomultiplier PMT + 500nm (Hamamatsu, R928P) detector. Quantum yields ($\Phi_{PL}$) measurements were performed using Avantes AvaSphere-80 integrating sphere (Edinburgh Instruments) and absolute method.

Films, 0.01g of compound in 0.5cm$^{3}$ of solvent, were fabricated from a homogenous chloroform solution. The obtained mixtures were spin-coated on glass substrates. Films were dried for 20h in a vacuum oven at 60°C.

The morphology of the prepared layers was characterized by atomic force microscopy (AFM) using a TopoMetrix Explorer device, operating in contact mode, in air, in the constant force regime. The films doped with lithium salt with the highest PCE were tested.

The cross-sectional SEM images were taken using the SEM microscope Quanta/FEG 250/FEI Company. FTO/b-$TiO_2$/m-$TiO_2$/perovskite/HTM films analogous to the devices were prepared by a method identical to that used in PSCs fabrications and I-V measurements.

The fluorine-doped tin oxide coated glass slides were cleaned with surfactant, hot deionized water and isopropanol in an ultrasonic bath. Subsequently, a blocking layer b-$TiO_2$ and mesoporous m-$TiO_2$ layer were prepared on FTOs as described in the paper [1]. Perovskite layers were obtained by the two-step method. The mixture of the oxetanes so-

lution was 36.15 mg of the oxetanes in 0.5 mL of chlorobenzene, 14.4μL of 4-tert-butyl pyridine, and 8.75μL of lithium bis(trifluoromethanesulfonyl)imide (260 mg Li-TFSI in 0.5 mL acetonitrile). The foregoing oxetanes solution was spin-coated on the FTO/b-$TiO_2$/m-$TiO_2$ at 4.000 rpm for 30 s [1]. The gold electrode was deposited by thermal evaporation~$10^{-6}$mbar on the FTO/b-$TiO_2$/m-$TiO_2$/perovskite/HTM. The fabricated devices were examined using the PET Photo Emission Tech Inc. Model SS 200AAA class solar simulator in Standard Test Conditions (STC) (active area 0.25 $cm^2$, 25 °C, 1000 $W/m^2$, AM1.5).

The theoretical calculations were performed with the use of the density functional theory (DFT) and were carried out using the Gaussian09 program [3] on B3LYP/6-311g++ level [3,4]. Molecular geometry of the singlet ground state of the compounds was optimized in the gas phase and the frequency calculation for each of the compounds shows only positive values, which verifies that the optimized molecular structure corresponds to the energy minimum. Solvent effect was taken into account using the polarizable continuum model (PCM) [4] with dichloromethane and chloroform as solvents. Such calculations were carried out for analysis of the frontier molecular orbital structure, energy levels and UV–Vis data. The optimized geometries of the compounds are depicted in Fig. S12, and Fig. S11 presents experimental and calculated IR spectra. Density of states diagrams (Fig. S13) were obtained with use of the GaussSum program [5]. Calculated LUMOs energies were overestimated but the calculated values of the HOMO and LUMO energies were used only for consistency with geometry optimization. Moreover the TD-DFT method [6] was employed to calculate the geometries in first singlet and triplet excited states. Figure S12a presents the relative geometries of ground and first singlet and triplet excited states.

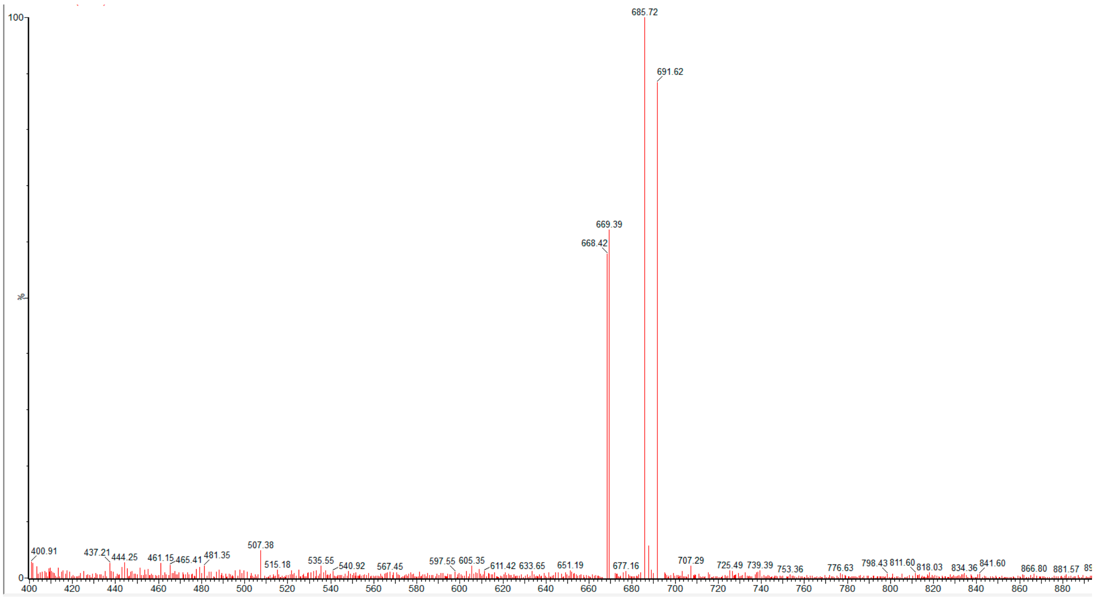

**Mass spectrum of compound 4.**

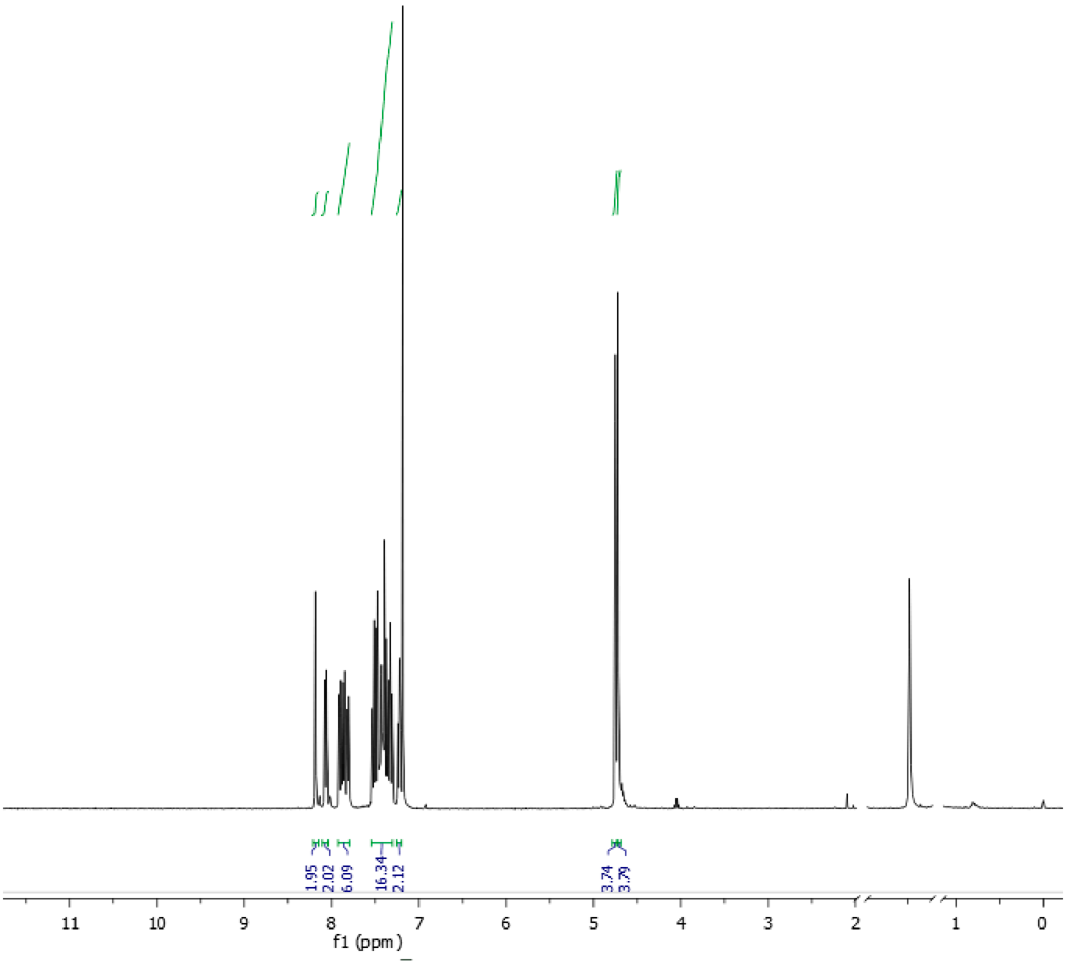

**$^1$H NMR spectrum of compound 4.**

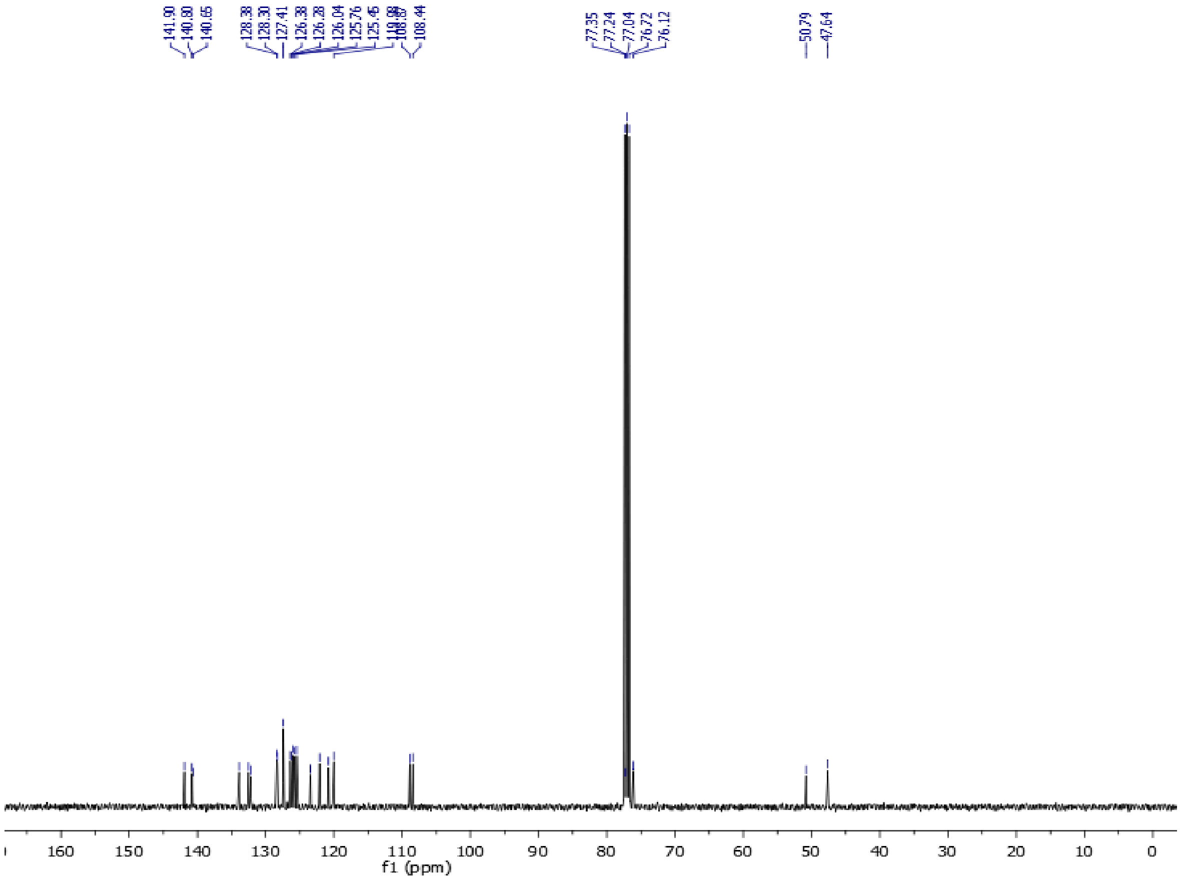


**$^{13}C$ NMR spectrum of compound 4.**

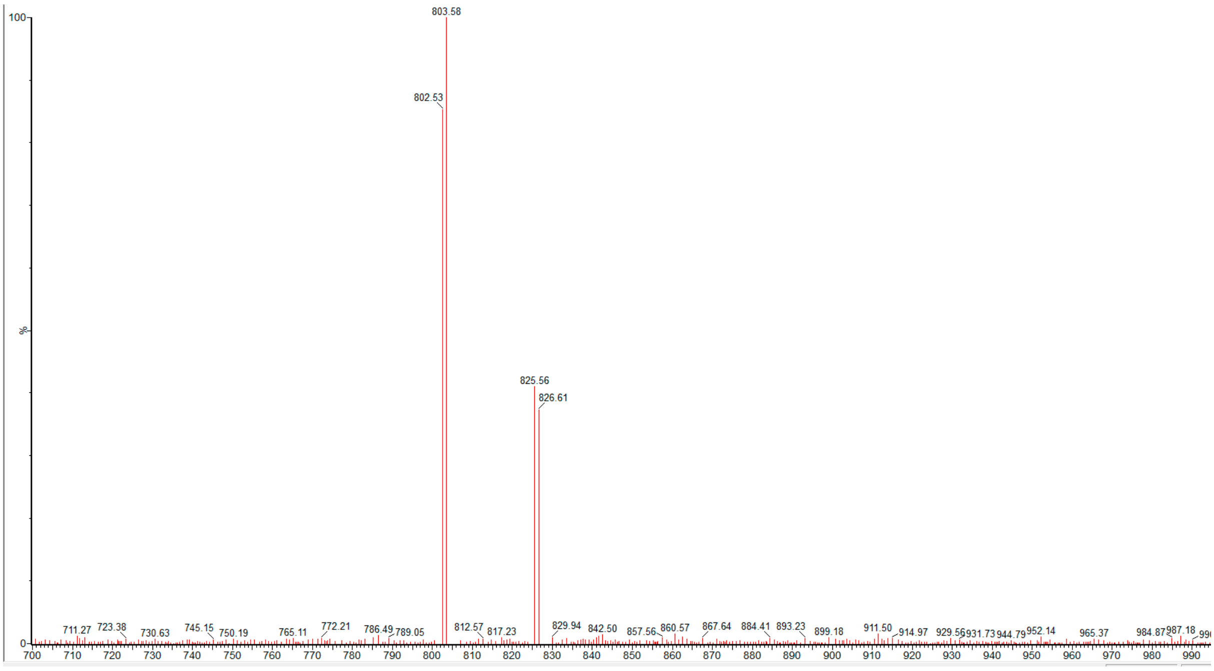

**Mass spectrum of compound 5.**

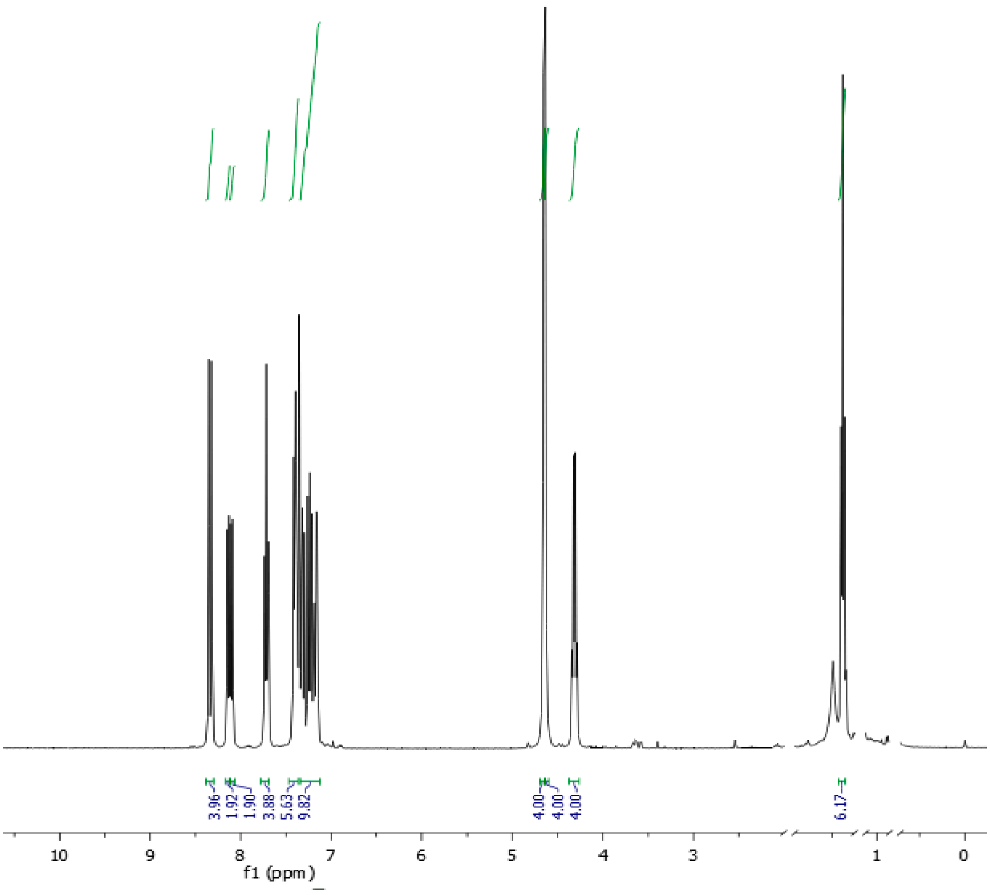


**$^{1}$H NMR spectrum of compound 5.**

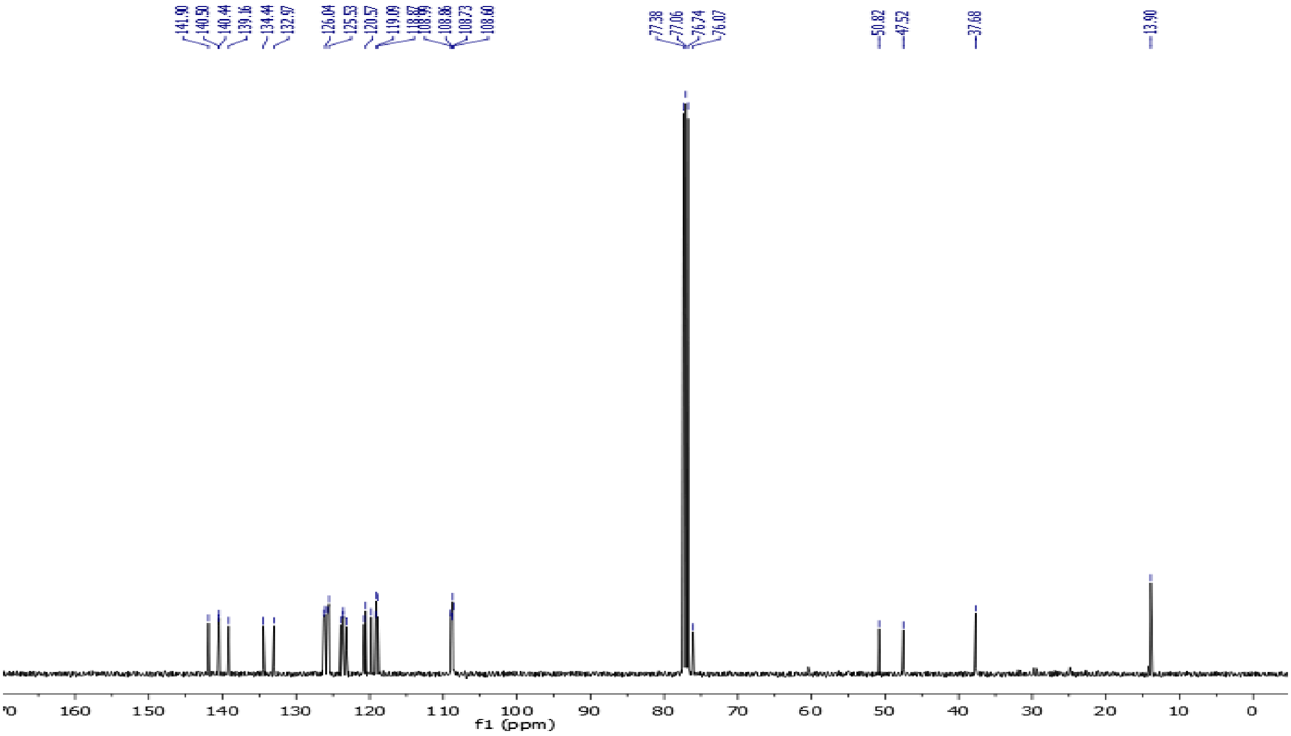


**$^{13}$C NMR spectrum of compound 5.**

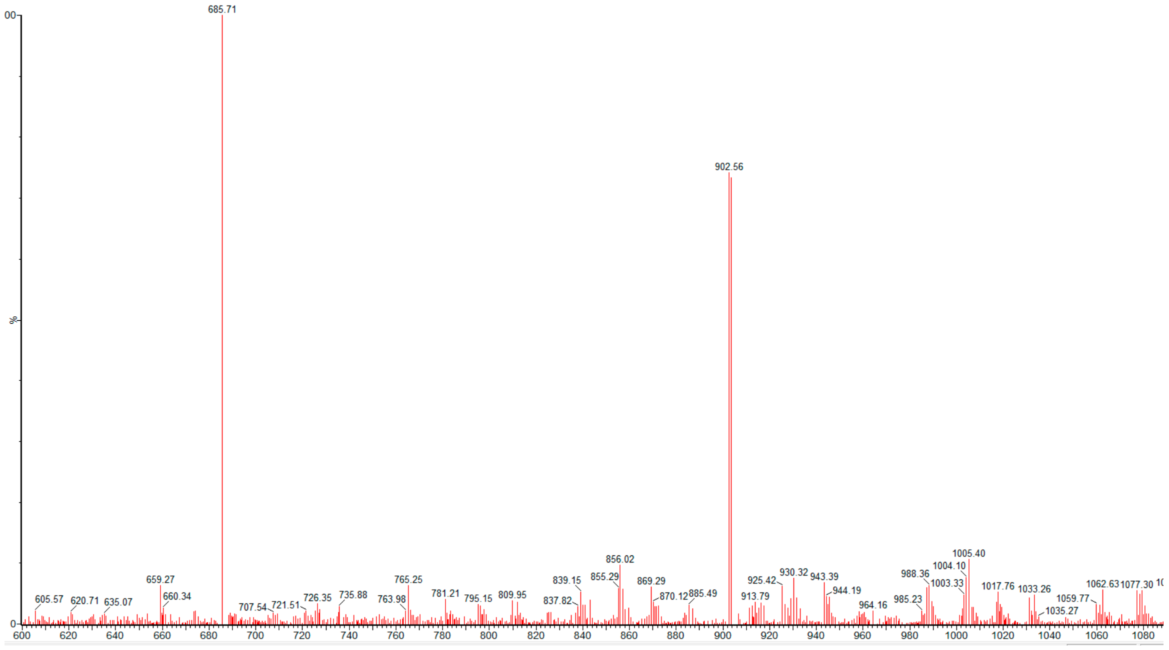


**Mass spectrum of compound 6.**

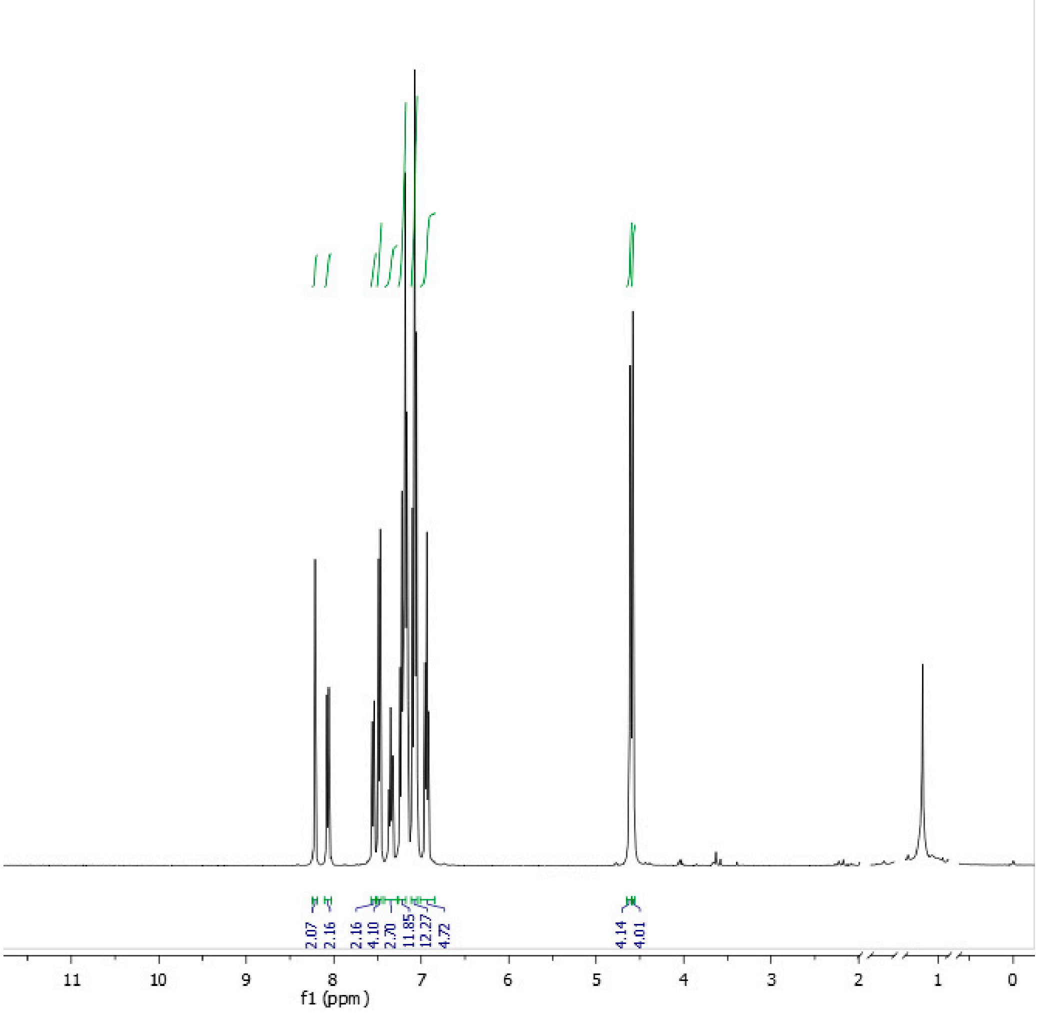


**$^{1}$H NMR spectrum of compound 6.**

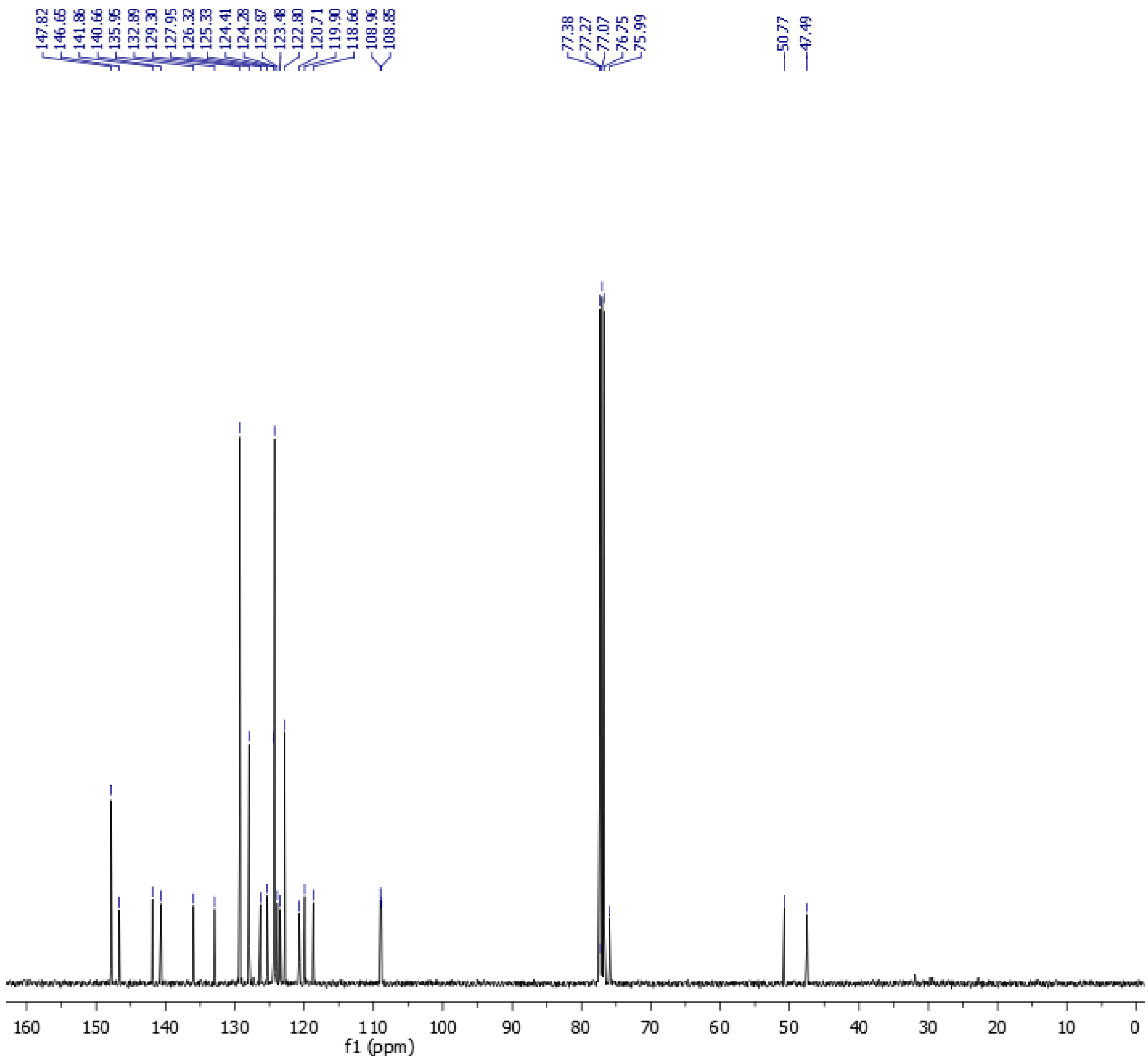


**$^{13}C$ NMR spectrum of compound 6.**

**Figure S1.** Mass spectra, $^{1}H$ NMR and $^{13}C$ NMR spectra of compounds **4**, **5** and **6**.

**Thermal properties**

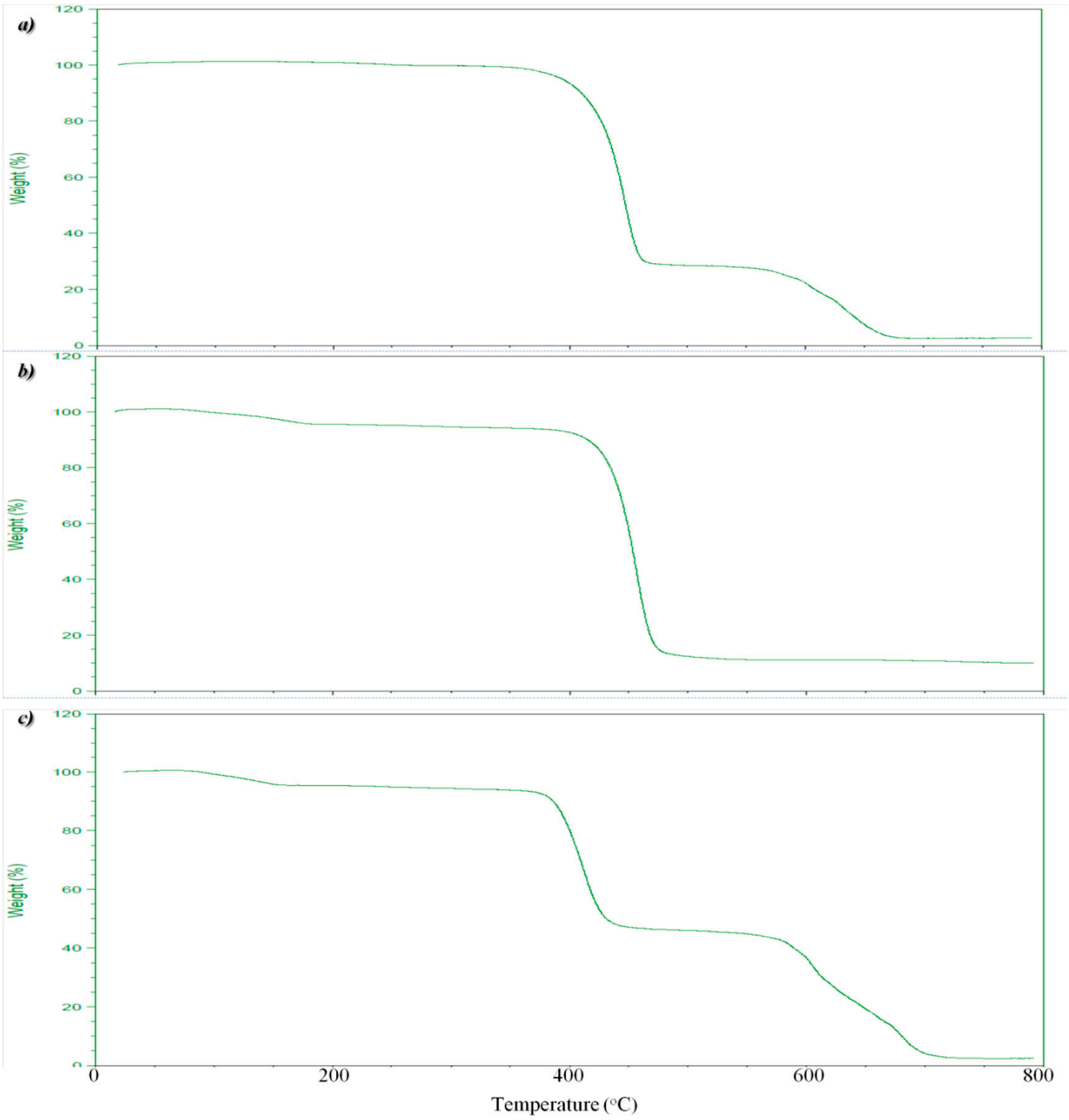


**Figure S2.** TGA curves of compounds: **a**) **4**, **b**) **5**, **c**) **6**.

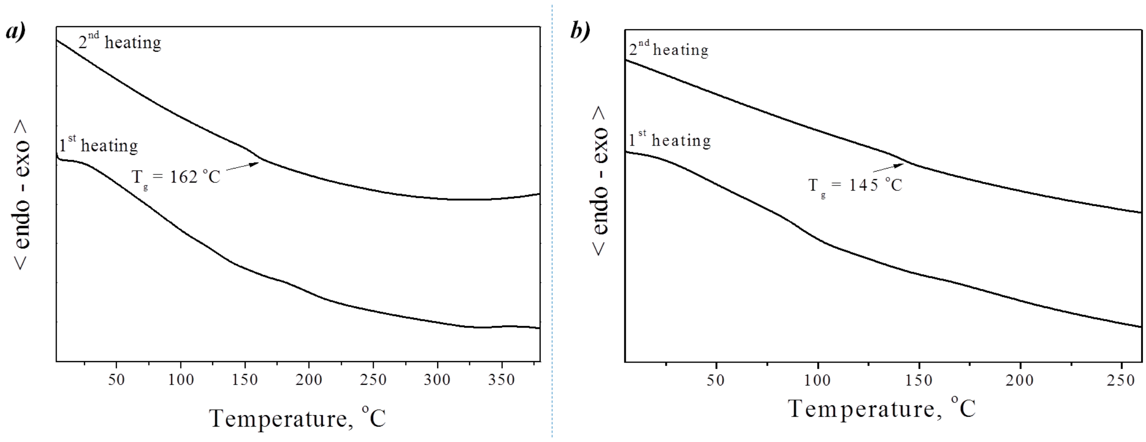


**Figure S3.** DSC curves of the compounds: **a**) **5**, **b**) **6**.

**Electrochemical properties**

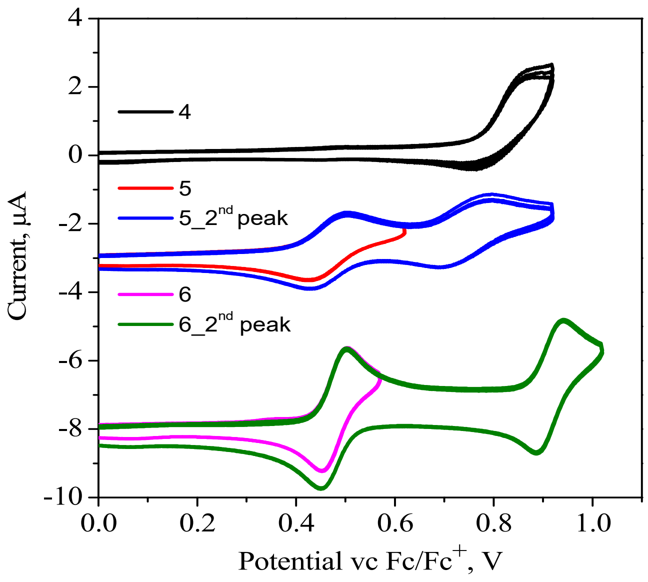


**Figure S4.** Additional scans for compounds **4**, **5** and **6**.

**Table S1.** Number of electrons during oxidation process.

| **Code** | **Number of Electrons** | |
|---|---|---|
| | **1st Oxidation Peak** | **2nd Oxidation Peak** |
| ***6*** | 1.04 | 1.00 |
| ***5*** | 1.00 | 1.03 |

**Table S2.** Composition of the selected molecular orbitals.

| | ***4*** | | | | ***5*** | | | | ***6*** | | | |
|---|---|---|---|---|---|---|---|---|---|---|---|---|
| | eV | oxime | carbazole | R | eV | oxime | carbazole | R | eV | oxime | carbazole | R |
| L+10 | 0.40 | 5 | 74 | 21 | 0.42 | 2 | 27 | 71 | -0.25 | 0 | 11 | 89 |
| L+9 | 0.08 | 2 | 72 | 26 | 0.13 | 1 | 43 | 55 | -0.27 | 0 | 6 | 94 |
| L+8 | 0.00 | 5 | 77 | 19 | 0.05 | 4 | 64 | 31 | -0.27 | 0 | 8 | 92 |
| L+7 | -0.40 | 0 | 80 | 20 | -0.12 | 1 | 61 | 38 | -0.74 | 0 | 0 | 100 |
| L+6 | -0.41 | 0 | 80 | 20 | -0.24 | 0 | 16 | 83 | -0.74 | 0 | 0 | 100 |
| L+5 | -0.67 | 0 | 6 | 94 | -0.38 | 0 | 60 | 39 | -0.87 | 0 | 1 | 99 |
| L+4 | -0.67 | 0 | 6 | 94 | -0.73 | 0 | 52 | 47 | -0.87 | 0 | 1 | 99 |
| L+3 | -1.30 | 1 | 88 | 11 | -1.22 | 0 | 2 | 98 | -1.08 | 0 | 34 | 66 |
| L+2 | -1.32 | 1 | 86 | 12 | -1.28 | 1 | 97 | 2 | -1.10 | 1 | 34 | 66 |
| L+1 | -1.50 | 0 | 16 | 84 | -1.30 | 1 | 84 | 15 | -1.31 | 1 | 95 | 3 |
| LUMO | -1.51 | 0 | 16 | 84 | -1.37 | 0 | 31 | 69 | -1.33 | 2 | 96 | 2 |
| HOMO | -5.78 | 4 | 73 | 23 | -5.49 | 2 | 41 | 58 | -5.26 | 1 | 19 | 80 |
| H-1 | -5.84 | 3 | 67 | 30 | -5.67 | 3 | 60 | 38 | -5.29 | 0 | 14 | 86 |
| H-2 | -6.18 | 1 | 61 | 38 | -5.78 | 0 | 1 | 99 | -5.86 | 6 | 71 | 22 |
| H-3 | -6.20 | 1 | 67 | 31 | -6.03 | 2 | 45 | 53 | -6.03 | 3 | 77 | 20 |
| H-4 | -6.44 | 0 | 61 | 39 | -6.20 | 1 | 51 | 48 | -6.30 | 0 | 96 | 4 |
| H-5 | -6.44 | 1 | 62 | 37 | -6.22 | 2 | 71 | 27 | -6.30 | 0 | 95 | 5 |
| H-6 | -7.04 | 0 | 1 | 99 | -6.35 | 0 | 63 | 37 | -6.99 | 34 | 26 | 40 |
| H-7 | -7.04 | 0 | 1 | 99 | -6.44 | 1 | 62 | 37 | -7.03 | 1 | 44 | 56 |
| H-8 | -7.19 | 81 | 18 | 1 | -7.09 | 1 | 46 | 54 | -7.10 | 58 | 15 | 27 |
| H-9 | -7.32 | 1 | 89 | 10 | -7.17 | 78 | 16 | 6 | -7.13 | 0 | 0 | 100 |
| H-10 | -7.36 | 14 | 76 | 10 | -7.26 | 2 | 1 | 97 | -7.13 | 0 | 0 | 100 |

## Spectroscopic Studies

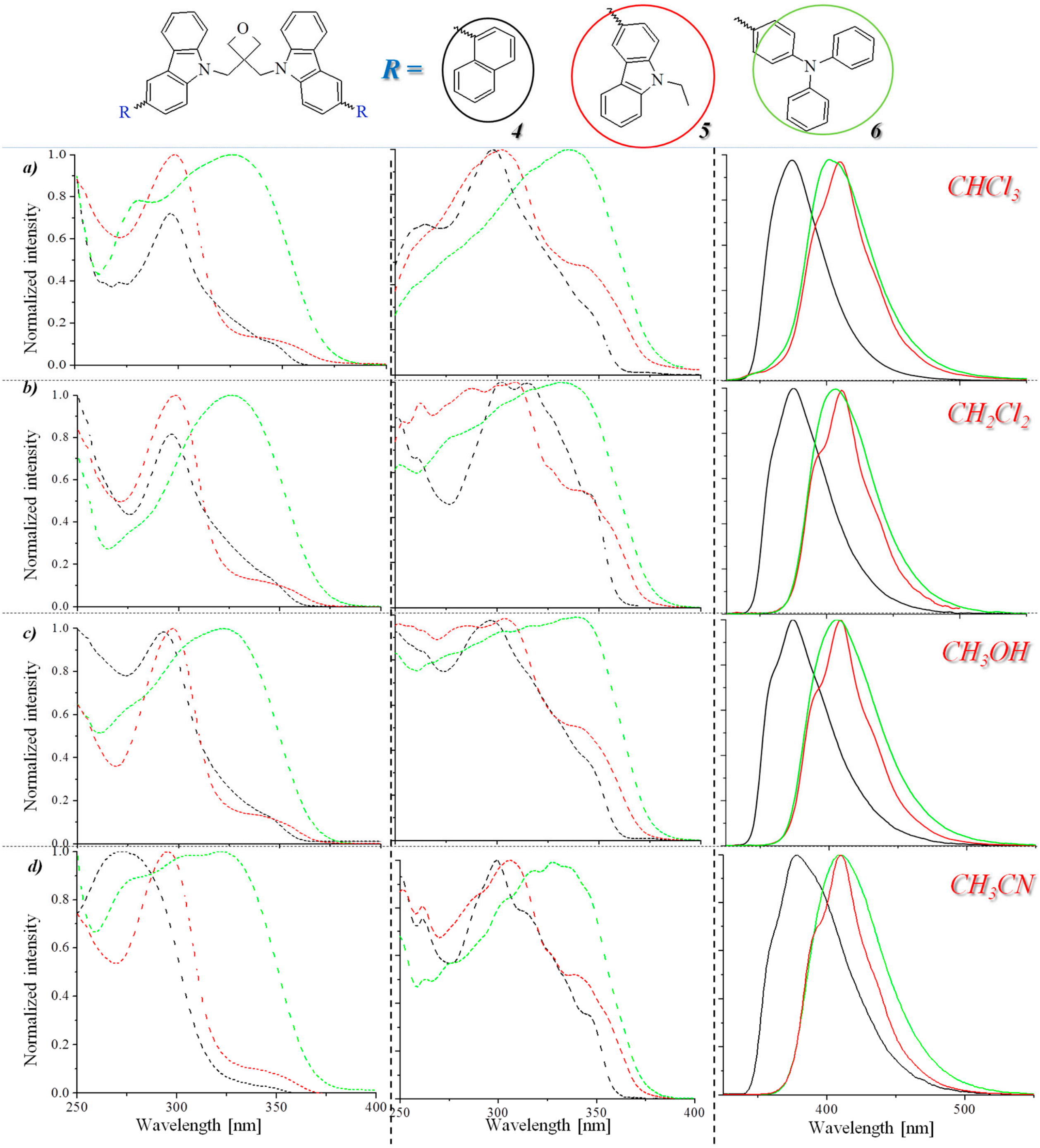


**Figure S5.** Imposed UV–Vis and excitation spectra, respectively (*dotted line*) as well as PL spectra (*solid line*) of compounds (**4**, **5** and **6**) in various solvents: a) chloroform ($CHCl_3$), b) dichloromethane ($CH_2Cl_2$), c) methanol ($CH_3OH$), d) acetonitrile ($CH_3CN$).

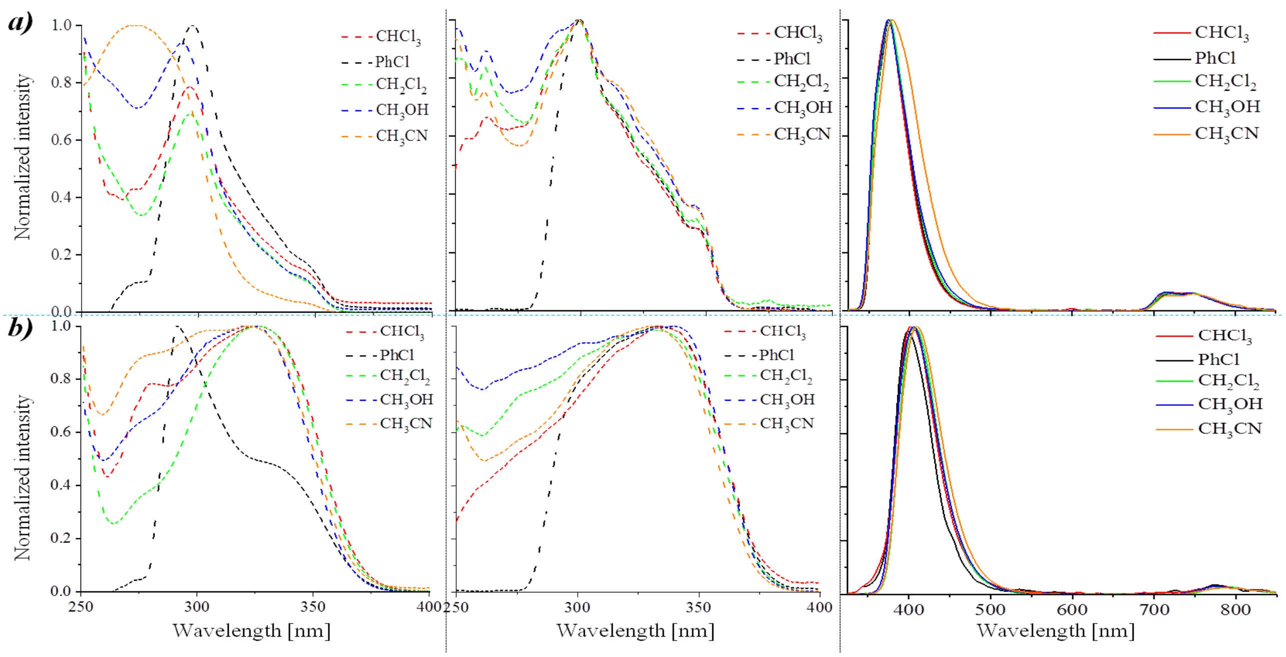


**Figure S6.** Imposed UV–Vis and excitation spectra, respectively (dotted line) as well as PL spectra (solid line) in various solvents for compound: a) ***4***, b) ***6***.

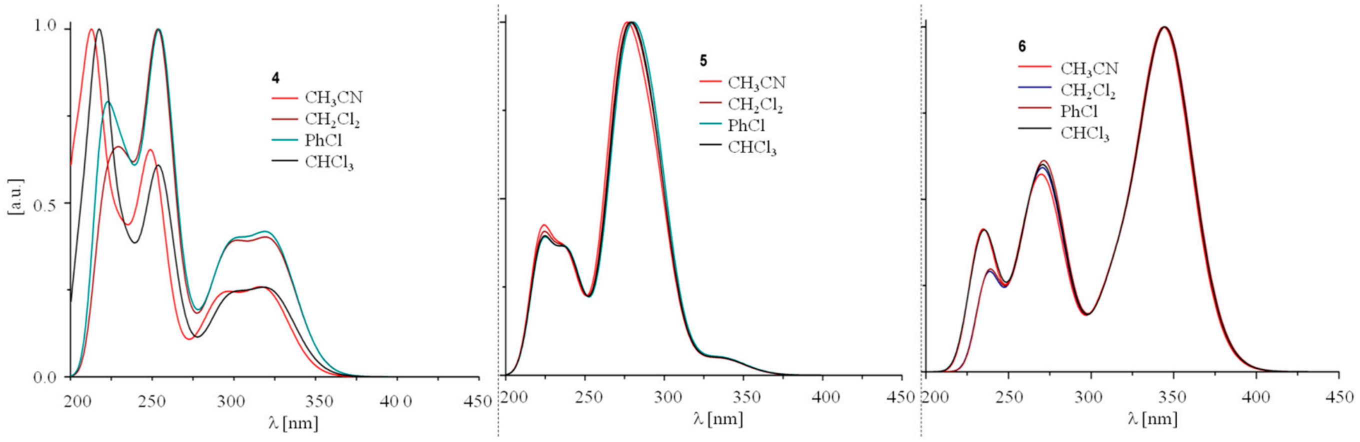


**Figure S6a.** Calculated electronic absorption spectra of compounds 4, 5 and 6 in selected solvents.

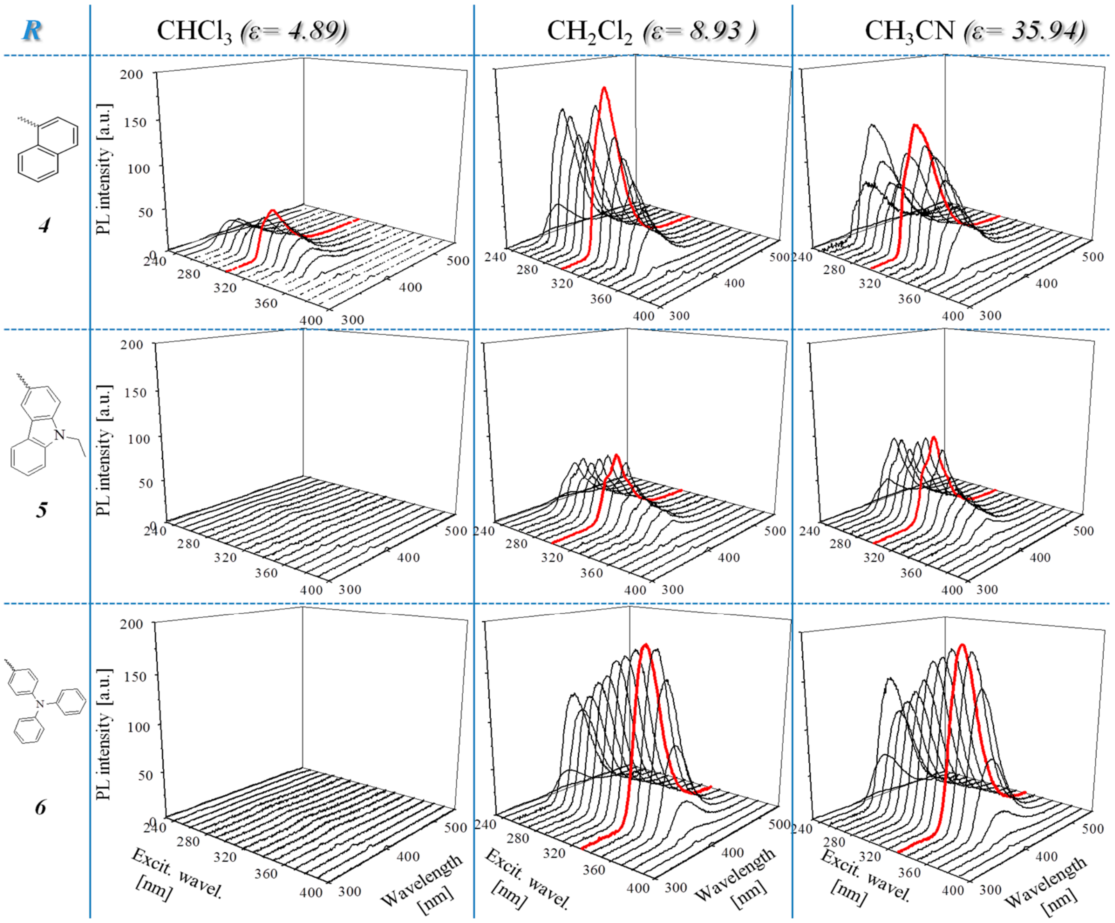


**Figure S7.** 3D spectra for the analyzed compounds (***4***, ***5***, ***6***) in the excitation range from 230 to 400 nm and the collected emissions in the range from 300 to 550 nm in three solvents: chloroform (***CHCl₃***), dichloromethane (***CH₂Cl₂***) and acetonitryl (***CH₃CN***).

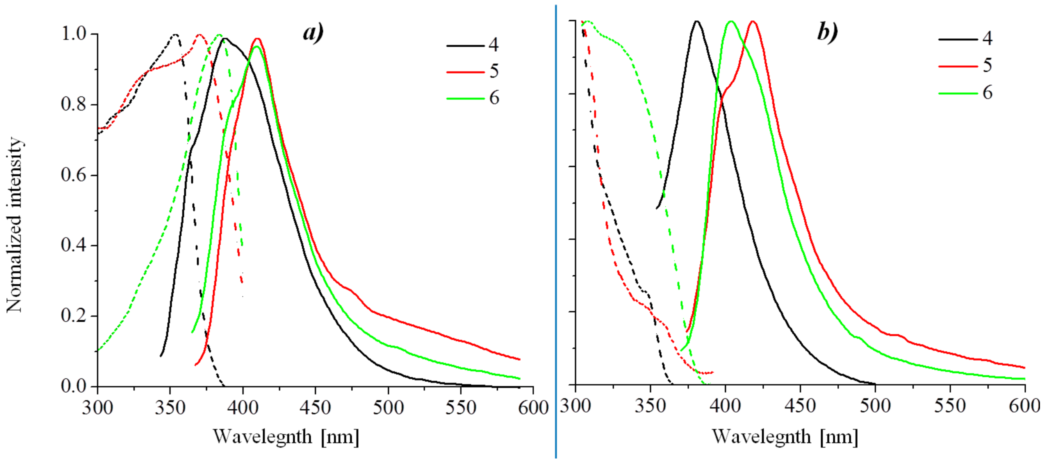


**Figure S8.** The excitation (*dotted line*) PL spectra (*solid line*) for compounds in a) powder, b) film.

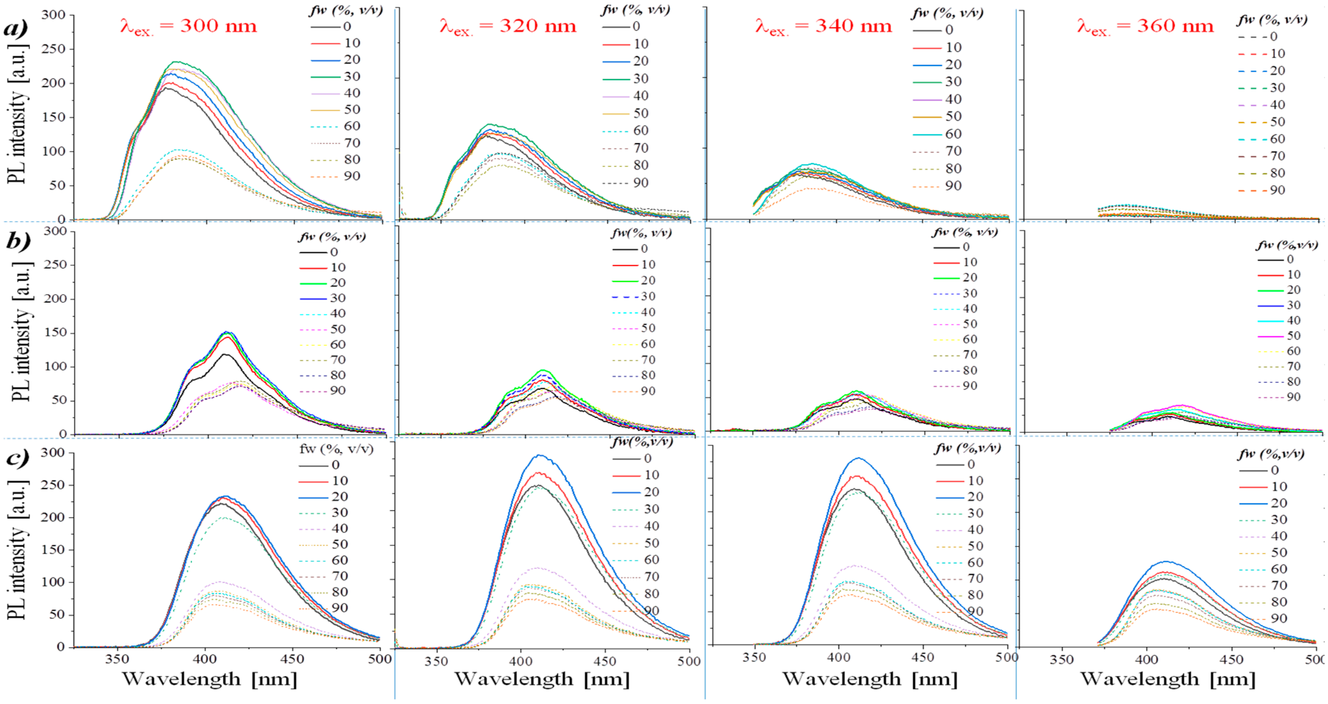


**Figure S9.** The photoluminescence (PL) properties of tested compounds (*10 μM concentration*) in a binary mixture of $CH_3CN/H_2O$ with an increasing water content (***fw*** = 0, 10, 20, 30, 40, 50, 60, 70, 80, 90 %, v/v) at different excitation wavelengths ( $_{ex.}$: 300, 320, 340, 360 nm) for: a) ***4***, b) ***5***,c) ***6***.

| Device structure | $V_{Li\text{-}TFSI}$ [μL] | Current [mA] - Voltage [V] | Current Density [mA/cm²] - Voltage [V] |
|---|---|---|---|
| FTO/b-$TiO_2$/m-$TiO_2$/ perovskite/Au | - | 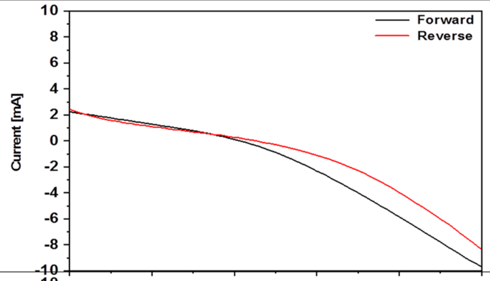 | 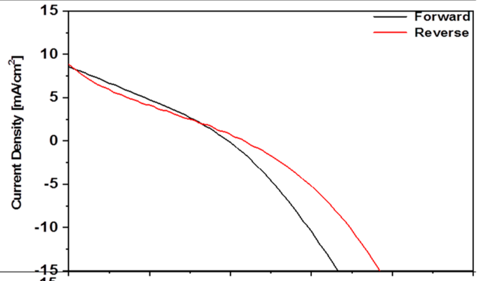 |
| | 8.75 | 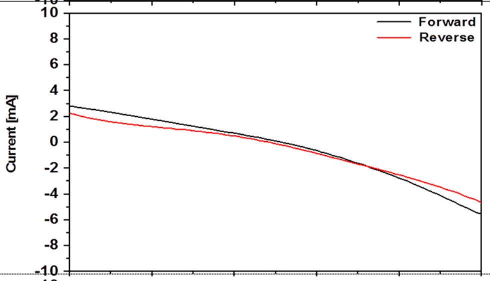 | 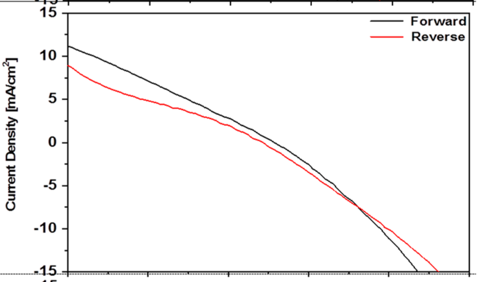 |
| FTO/b-$TiO_2$/m-$TiO_2$/ perovskite/4/Au | 17.50 | 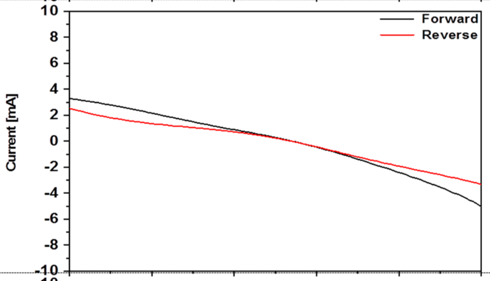 | 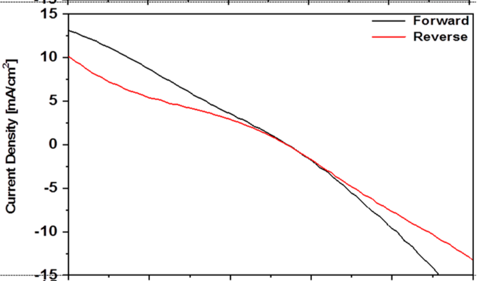 |
| | 35. 00 | 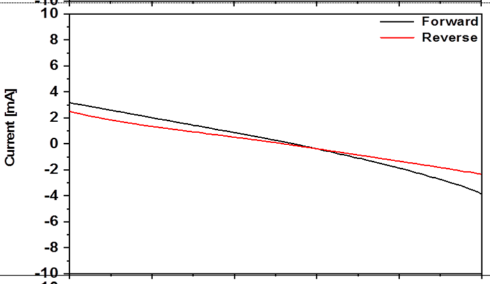 | 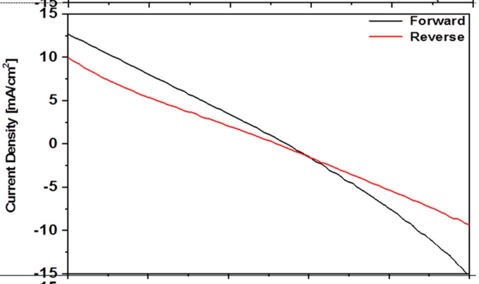 |
| FTO/b-$TiO_2$/m-$TiO_2$/ perovskite/5/Au | 8.75 | 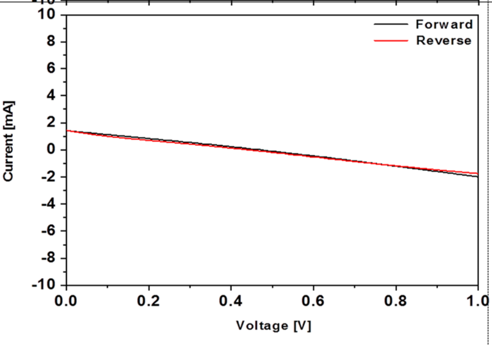 | 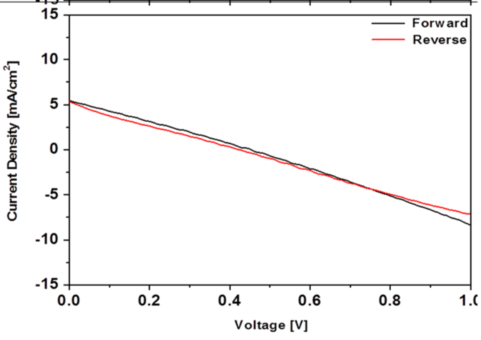 |

**Figure S10.** I-V characteristics of the champion devices.

| Device structure | $V_{Li\text{-}TFSI}$ [µL] | Current [mA] - Voltage [V] | Current Density [mA/cm²] - Voltage [V] |
|---|---|---|---|
| FTO/b-$TiO_2$/m-$TiO_2$/perovskite/4/Au | 17.50 | 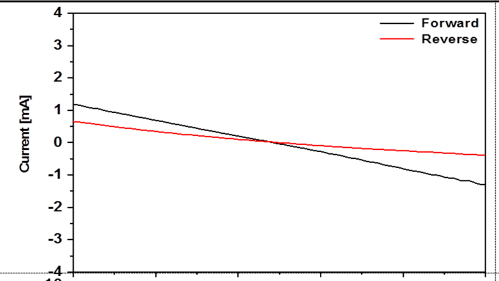 | 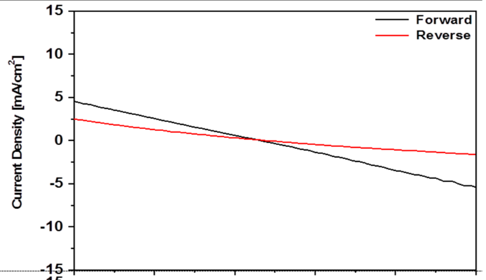 |
| | 35. 00 | 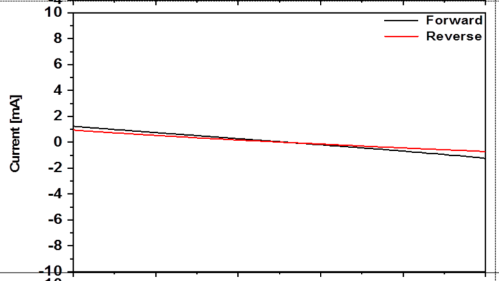 | 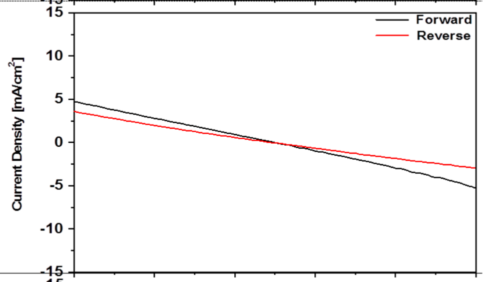 |
| FTO/b-$TiO_2$/m-$TiO_2$/perovskite/6/Au | 8.75 | 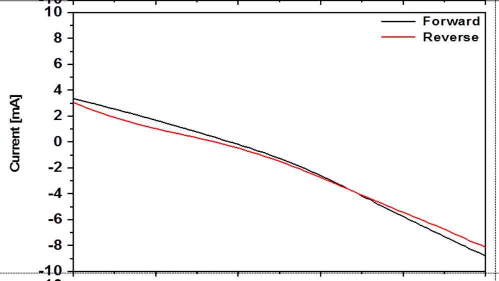 | 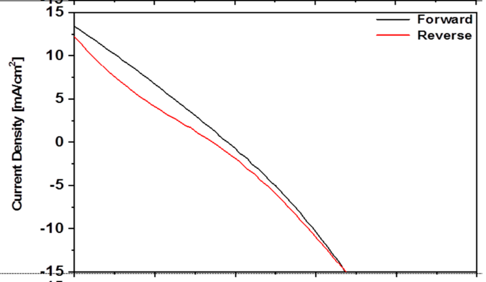 |
| | 17.50 | 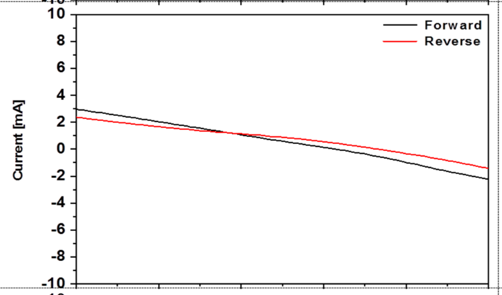 | 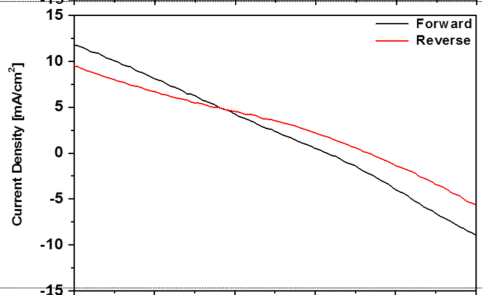 |
| | 35. 00 | 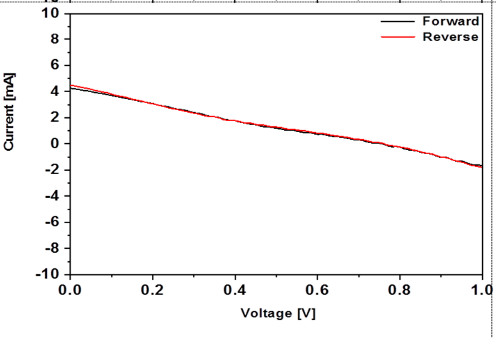 | 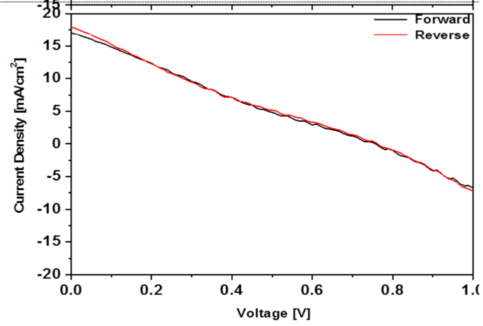 |

**Figure S10.** ***(continued)*** I-V characteristics of the champion devices.

## Theoretical calculation

**Table S3.** Energy differences [nm] between ground and $S_1$, $T_1$ excited states calculated in $CH_2Cl_2$ solvent (the maxima of emission wavelength are given in parenthesis) and calculated dipole moments.

| | $E_{S1-S0}$ | $E_{T1-S0}$ | Dipole Moment [D] | | |
|---|---|---|---|---|---|
| | | | $S_0$ | $S_1$ | $T_1$ |
| **4** | **363 (374)** | 623 (714) | 2.85 | 3.54 | 3.38 |
| **5** | 437 (410) | 682 (778) | 3.60 | 3.63 | 3.23 |
| **6** | 385 (406) | 658 (782) | 4.24 | 4.49 | 4.29 |

**Table S4.** The calculated electronic transitions corresponding to excitation wavelength in $CH_2Cl_2$ solution.

| | exp | calc (*f*)# | Transition | Character |
|---|---|---|---|---|
| **4** | 300 | 305.2 (0.0193) | H–1→LUMO (33%); H–1→L+1 (24%); HOMO→LUMO (19%) | carbazole → R* |
| **5** | 306 | 309.7 (0.0467) | H-2→LUMO (65%) | carbazole → carbazole* |
| **6** | 330 | 334.1 (0.0564) | HOMO→L+3 (82%) | carbazole (41%) + R (58%)→ R* |

# taken into account were the calculated transitions at the wavelength closest to the experimental data with the highest transition coefficient in this energy range.

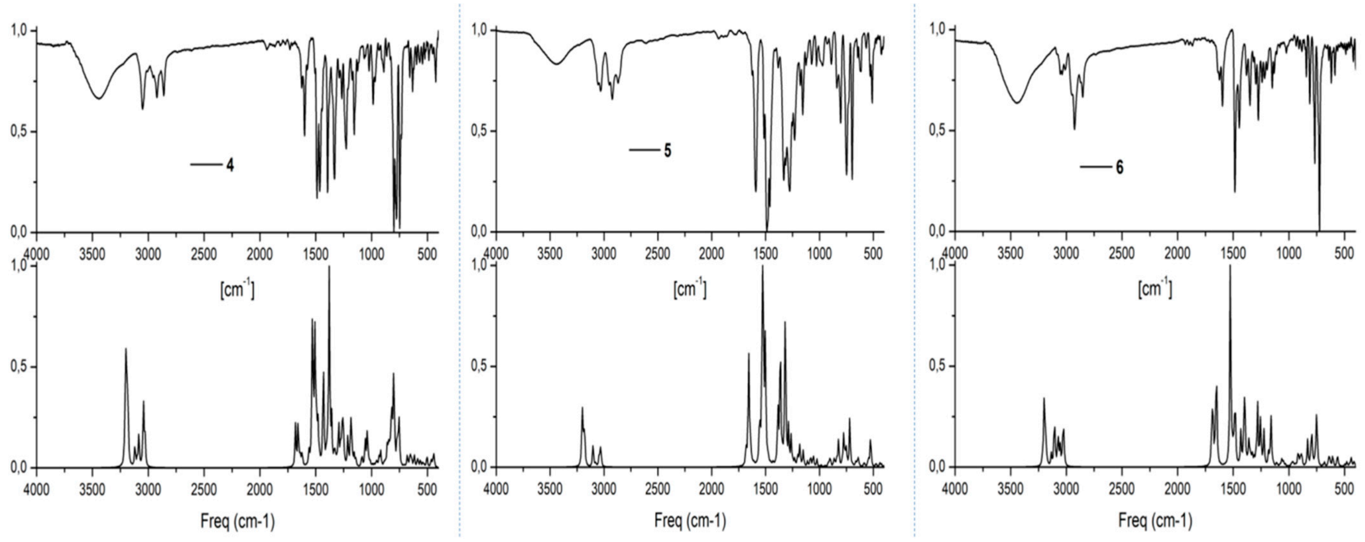


**Figure S11.** Experimental and calculated IR spectra of **4**, **5** and **6** compounds.

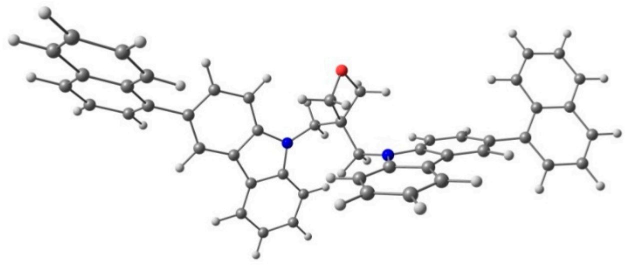

*4*

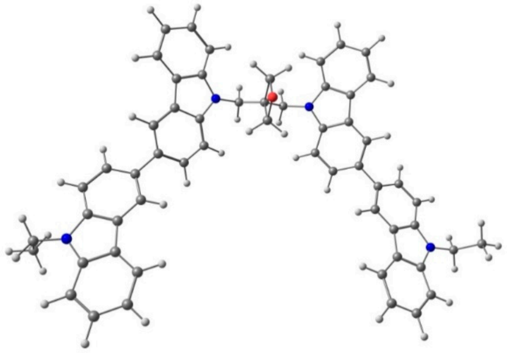

*5*

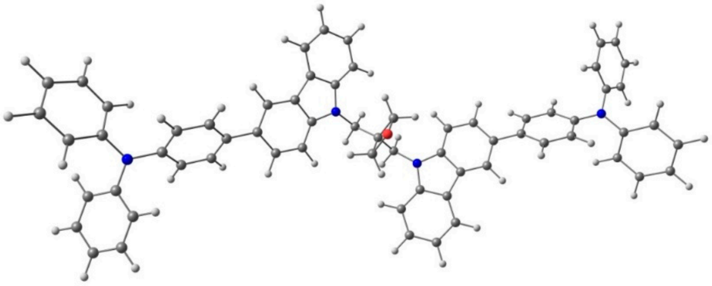

*6*

**Figure S12.** The optimized geometries of compounds **4**, **5** and **6**.

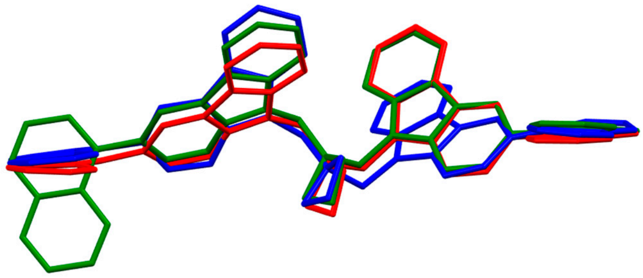

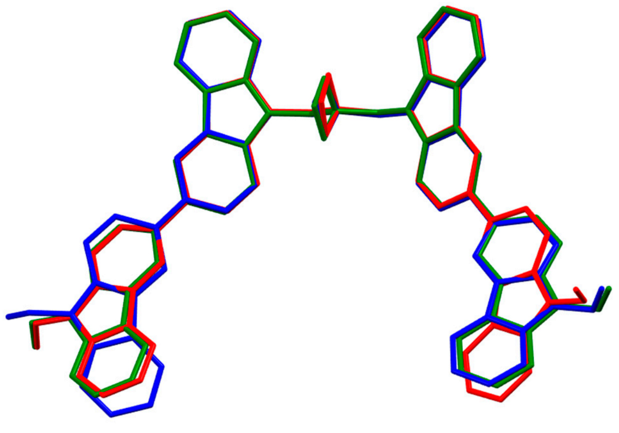

**4** **5**

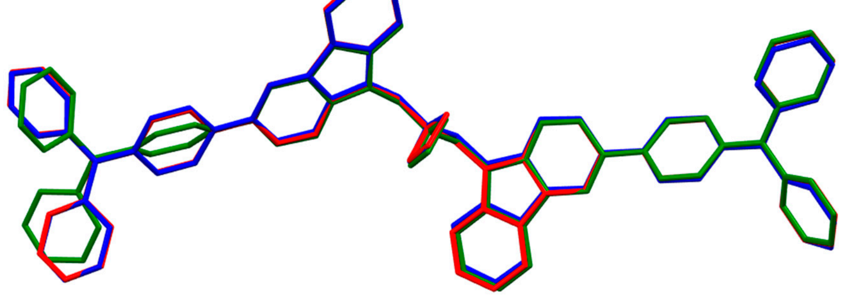

**6**

**Figure S12a.** Optimized geometries of ground (green), $S_1$ (red) and $T_1$ (blue) states of compounds **4**, **5** and **6**. (Hydrogen atoms were omitted for clarity).

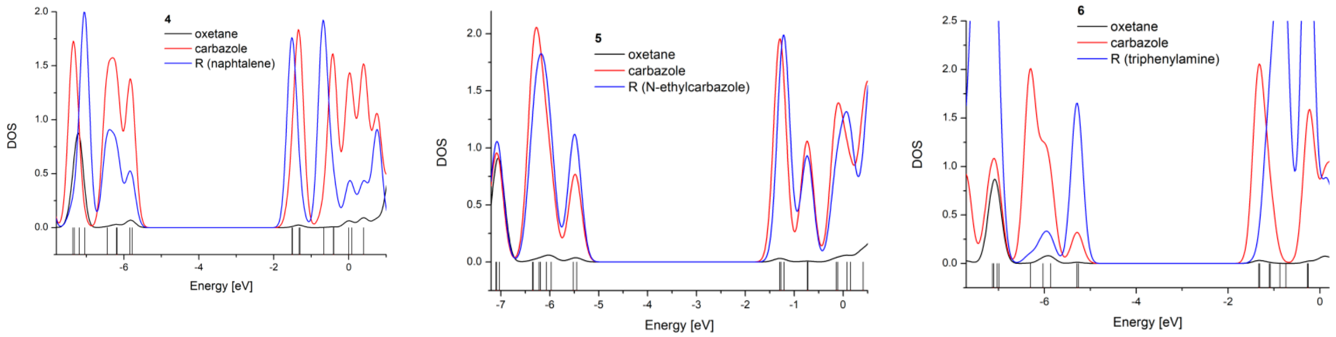


**Figure S13.** DOS spectra of the investigated compounds.

## References

1. Pająk A.K., Gnida P., Kotowicz S., Małecki J. G., Libera M., Bednarczyk K., Schab-Balcerzak E., New thiophene imines acting as hole transporting materials in photovoltaic devices, *Energy Fuels*, **2020**, *34*, 10160–10169.
2. Gaussian 09, RevisionA.02, Frisch M. J., Trucks G. W., Schlegel H. B., Scuseria G. E., Robb M. A., Cheeseman J. R., Scalmani G., Barone V., Petersson G. A., Nakatsuji H., Li X., Caricato M., Marenich A., Bloino J., Janesko B. G., Gomperts R., Mennucci B., Hratchian H. P., Ortiz J. V., Izmaylov A. F., Sonnenberg J. L., Williams-Young D., Ding F., Lipparini F., Egidi F., Goings J., Peng B., Petrone A., Henderson T., Ranasinghe D., Zakrzewski V. G., Gao J., Rega N., Zheng G., Liang W., Hada M., Ehara M., Toyota K., Fukuda R., Hasegawa J., Ishida M., Nakajima T., Honda Y., Kitao O., Nakai H., Vreven T., Throssell K., Montgomery J. A. Jr., Peralta J. E., Ogliaro F., Bearpark M., Heyd J. J., Brothers E., Kudin K. N., Staroverov V. N., Keith T., Kobayashi R., Normand J., Raghavachari K., Rendell A., Burant J. C., Iyengar S. S., Tomasi J., Cossi M., Millam J. M., Klene M., Adamo C., Cammi R., Ochterski J. W., Martin R. L., Morokuma K., Farkas O., Foresman J. B., and D. J. Fox, gaussian 09, Revision d. 01, Gaussian., Gaussian, Inc., Wallingford CT, 2016.
3. Becke A. D., Density-functional thermochemistry. III. The role of exact exchange *J.Chem.Phys.* **1993**, 98, 5648–5652.
4. Lee C., Yang W., Parr R.G., Development of the Colle-Salvetti correlation-energy formula into a functional of the electron density *Phys. Rev.* **1988**, *B 37*, 785–789.
5. O'Boyle N.M., Tenderholt A.L., Langner K.M. Cclib: a library for package-independent computational chemistry algorithms *J. Comp. Chem*. **2008**, 29, 839-845.
6. M.E. Casida, in: J.M. Seminario (Ed.), Recent Developments and Applications of Modern Density Functional Theory, Theoretical and Computational Chemistry, vol. 4, Elsevier, Amsterdam, 1996, p. 391.
